# Genetic and correlative light and electron microscopy evidence for the unique differentiation pathway of erythrophores in brown trout skin

**DOI:** 10.1038/s41598-022-04799-7

**Published:** 2022-01-19

**Authors:** Simona Sušnik Bajec, Ida Djurdjevič, Carmen Linares Andújar, Mateja Erdani Kreft

**Affiliations:** 1 Department of Animal Science, Biotechnical Faculty, University of Ljubljana, Jamnikarjeva 101, 1000 Ljubljana, Slovenia; 2 Centro de Investigaciones, Biológicas Margarita Salas - CSIC, Calle Ramiro de Maeztu, 9, 28040 Madrid, Spain; 3 Institute of Cell Biology, Faculty of Medicine, University of Ljubljana, Vrazov trg 2, 1000 Ljubljana, Slovenia

**Keywords:** Gene expression profiling, Super-resolution microscopy, Development, Molecular evolution, Zoology, Single-cell imaging

## Abstract

Based on their cell ultrastructure, two types of erythrophores in the spotted skin regions of brown trout (*Salmo trutta*) were previously described. To test the hypothesis regarding the origin of a new cell type following genome duplication, we analysed the gene and paralogue gene expression patterns of erythrophores in brown trout skin. In addition, the ultrastructure of both erythrophore types was precisely examined using transmission electron microscopy (TEM) and correlative light microscopy and electron microscopy (CLEM). Ultrastructural differences between the sizes of erythrophore inclusions were confirmed; however, the overlapping inclusion sizes blur the distinction between erythrophore types, which we have instead defined as cell subtypes. Nevertheless, the red spots of brown trout skin with subtype 2 erythrophores, exhibited unique gene expression patterns. Many of the upregulated genes are involved in melanogenesis or xanthophore differentiation. In addition, *sox10*, related to progenitor cells, was also upregulated in the red spots. The expressions of paralogues derived from two genome duplication events were also analysed. Multiple paralogues were overexpressed in the red spots compared with other skin regions, suggesting that the duplicated gene copies adopted new functions and contributed to the origin of a new cell subtype that is characteristic for red spot. Possible mechanisms regarding erythrophore origin are proposed and discussed. To the best of our knowledge, this is the first study to evaluate pigment cell types in the black and red spots of brown trout skin using the advanced CLEM approach together with gene expression profiling.

## Introduction

Teleost fish exhibit a variety of skin pigmentations and patterning. This great diversity of pigments, types of pigment cells (i.e. chromatophores), and pigment pattern is influenced by duplicate copies of pigmentation genes^1,2^. At least six main chromatophores with specific pigments are present in teleost fish: (1) melanophores (melanin, black/brown), (2) xanthophores (pteridine pigment, yellow), (3) iridophores (colourless guanine crystals, reflective/iridescent), (4) erythrophores (carotenoid pigments, red), (5) leucophores (crystalline purines reflect light), and (6) cyanophores (undefined pigments, blue)^2^. Mosaic chromatophores, containing more than one pigment (e.g. erythro-iridophores or cyano-erythrophores), and additional new pigment cell types and subtypes are also being described^3–5^. Deciphering the underlying mechanisms of diverse pigment patterns and pigmentations in adult fish specimens using genetic and developmental analyses has been challenging^2^. The list of genes known to affect pigmentation in teleost fish is constantly increasing but has mainly focused on skin pigmentation-related processes in zebrafish (*Danio rerio*)^6,7^.

Red pigmentation in teleost fish is caused by erythrophores, red–orange pigment cells containing carotenoid pigments. Together with melanophores, xanthophores, and iridophores, erythrophores were found in the skin of some adult Salmonidae fish species: *Salmo trutta* and *Salmo salar*^4,8^. They are distinguished from xanthophores based on the absence of xanthosomes, which are present only in xanthophores, while carotenoid-containing inclusions are present in both cell types, but may differ in size.

Using transmission electron microscopy (TEM), we recently described two types of erythrophores in the spotted skin regions of brown trout (*S. trutta*^4^). Erythrophores with similar ultrastructural features as xanthophores, but containing only carotenoid vesicles and no xanthosomes, were found in the black spots and were designated as type 1 erythrophores. Type 2 erythrophores were more distinctive; they were densely filled with one type of round organelle of much larger size than carotenoid vesicles (named erythrosomes) and located only in the red spots of brown trout skin^4^. While type 1 erythrophores were sparse in black spots, in which melanophores predominate, type 2 erythrophores represented almost the only cell type in red spots, with fibroblasts and some isolated melanophores above them. Interestingly, erythrophores were not observed in marble trout (*Salmo marmoratus*), a phylogenetically closely related but phenotypically distinct trout species without spots but containing xanthophores, melanophores, and iridophores in the dark skin regions. It was proposed that melanophore-xanthophore and melanophore-erythrophore interactions play an important role in pigment pattern formation in trout^4^.

In brown trout, type 1 erythrophores and melanophores are found in black spots, and type 2 erythrophores interact with each other in red spots. All three predominant pigment cells in the spots (melanophores and two types of erythrophores) are dendritic cells specialised not only in the storage but also in the translocation of their numerous light-absorbing pigment organelles. Some studies even indicate evolutionary relationships between these cells (see^8^ and the “Discussion” for more details).

Recently, we revealed the expression of different biological/metabolic pathways in brown and marble trout skin as well as the differential expression of differently pigmented skin regions in brown trout using skin transcriptomic data^9^. Our most outstanding finding was the expression profile in the red spots, composed almost exclusively of type 2 erythrophores in the *stratum compactum*. Namely, numerous genes found to be overexpressed in brown trout skin compared with marble trout skin were in fact highly overexpressed in the red spots of brown trout skin (such as *mitf, mc1r, sox10*, and *ednrb*). This demonstrates the existence of specific chromatophore cell types, subtypes, or states, and/or surrounding cells in red spots that may be involved in metabolic pathways or the specific communication between chromatophores and their surrounding cells^9^. Since many of these upregulated genes are involved in melanogenesis, we hypothesise that these genes might be involved in the origin of new cell types, subtypes, or states in brown trout. Genome duplication-derived paralogue gene copies might play an important role in this process.

The aim of this study was to further analyse the gene expression pattern of erythrophores in brown trout skin and to test the hypothesis regarding the origin of new cell types following genome duplication. Therefore, a new set of candidate differentially expressed genes (DEGs; as defined from skin transcriptome analyses^9^) in the red spots were analysed, and their expression in the different pigmented skin regions in brown trout were compared. In addition, paralogue-specific expression of a set of DEGs was analysed, and different scenarios regarding erythrophore origin are proposed and discussed. To further and more precisely describe the ultrastructure of both erythrophore types in brown trout skin, TEM of the black and red spots in situ was also performed. Moreover, correlative light and electron microscopy (CLEM) was performed on both erythrophore types to unequivocally determine and compare their ultrastructure. To the best of our knowledge, this is the first study to evaluate pigment cell types in the black and red spots of brown trout skin using the advanced CLEM approach together with gene expression profiling.

## Results

### TEM and CLEM

**Figure 1:**
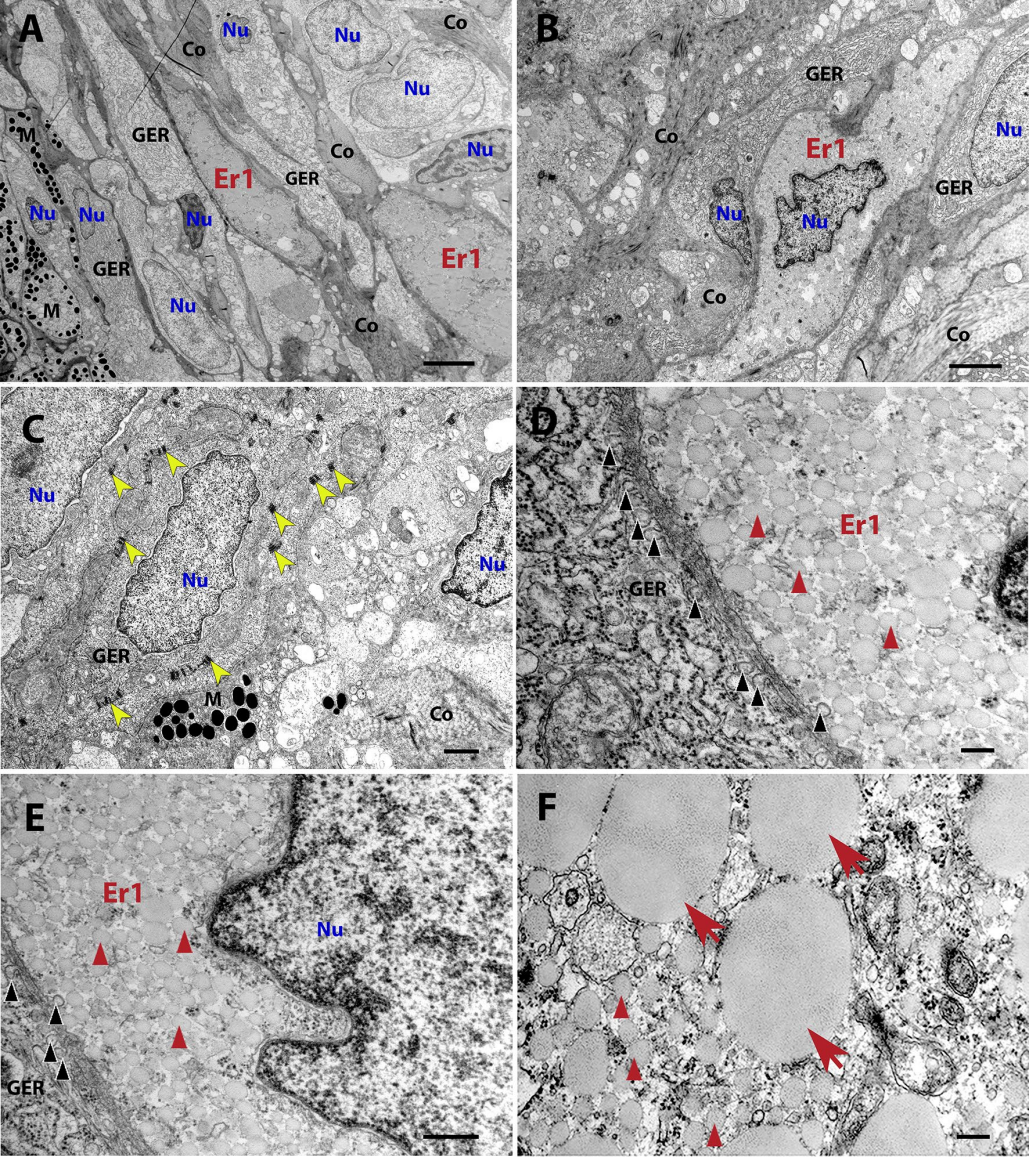
TEM images showing type 1 erythrophores from black spots. (**A**) A low magnification image of the dermis. Melanophores are positioned directly under the epidermis (on the left side). Type 1 erythrophores lie beneath melanophores. (**B**) Type 1 erythrophores surrounded by cells filled with closely packed sheets of GER and extracellular matrix with abundant collagen fibres (Co). (**C**) Desmosomes (yellow arrowheads) connecting the cells in the dermis. (**D**–**E**) Higher magnification images of areas from (**B**), showing the ultrastructure of type 1 erythrophore. Black arrowheads point to many caveolae between type 1 erythrophores and GER-filled cells (fibroblasts). Red arrowheads point to carotenoid vesicles. (**F**) Ultrastructure of type 1 erythrophore with carotenoid vesicles (red arrowheads) and some bigger, erythrosome-like inclusions (red arrows), while red arrows point to bigger, erythrosome-like inclusions. *Co* collagen fibres, *Er1* type 1 erythrophore, *GER* granular endoplasmic reticulum, *M* melanophore, *Nu* nucleus. Scale bars: 4 μm (**A**); 2 μm (**B**); 1 µm (**C**); 200 nm (**D**,**F**); 400 nm (**E**).

**Figure 2:**
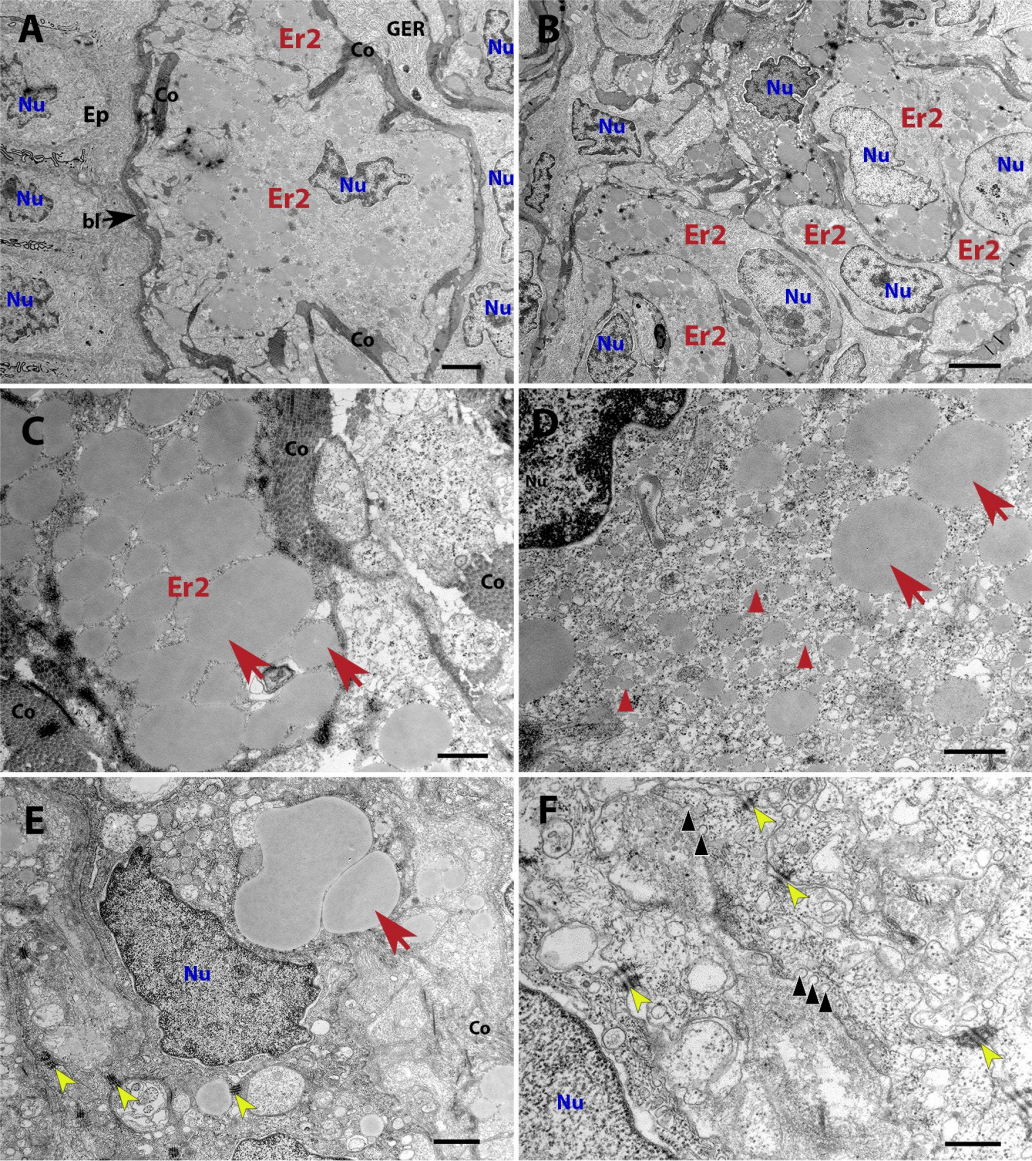
TEM images showing type 2 erythrophores from red spots. (**A**) A low magnification image of the area between the dermis and epidermis (on the left). (**B**) Type 2 erythrophores are the only pigment cells present in this skin area. (**C**–**E**) Ultrastructure of type 2 erythrophores, including erythrosomes (red arrows), carotenoid vesicles (red arrowheads). (**F**) Fibroblasts or fibroblast-like cells communicate with the extracellular space via prominent caveolae (black arrowheads) and are well connected with each other as well with type 2 erythrophores via desmosomes (yellow arrowheads),. *bl* basal lamina, *Co* collagen fibres, *Ep* epidermis, *Er2* type 2 erythrophore, *GER* granular endoplasmic reticulum, *Nu* nucleus. Scale bars: 2 μm (**A**); 4 μm (**B**); 600 nm (**C**,**D**); 1 μm (**E**); 400 nm (**F**).

In situ ultrastructural analyses of the centres of the black and red spots confirmed our previous data: the predominant chromatophores are melanophores and type 1 erythrophores in black spots and type 2 erythrophores in red spots. Based on the analysis of two independent red and two independent black spots, from which we altogether took 49 images, we further demonstrated that melanophores in black spots are positioned immediately under the epithelium and are separated from type 1 erythrophores by a layer of fibroblasts or fibroblast-like cells (Fig. 1A–D). Type 1 erythrophores have smaller carotenoid vesicles (207 ± 239 nm, Fig. 1D,E) and only occasionally larger erythrosome-like inclusions (up to 1 µm, Fig. 1F). In red spots, the predominant cells were type 2 erythrophores (Fig. 1A,B) with erythrosomes of 882 ± 270 nm in diameter (Fig. 2C,D), some with a diameter of up to 3.5 µm (Fig. 2E). It seems that type 2 erythrophores are stabilized in the centres of red spots by fibroblasts or fibroblast-like cells, which they communicate with the extracellular space via prominent caveolae and are well connected with each other as well with type 2 erythrophores via desmosomes (Fig. 2F). Both types of erythrophores are also surrounded by a prominent network of collagen fibres (Figs. 1 and 2).

**Figure 3:**
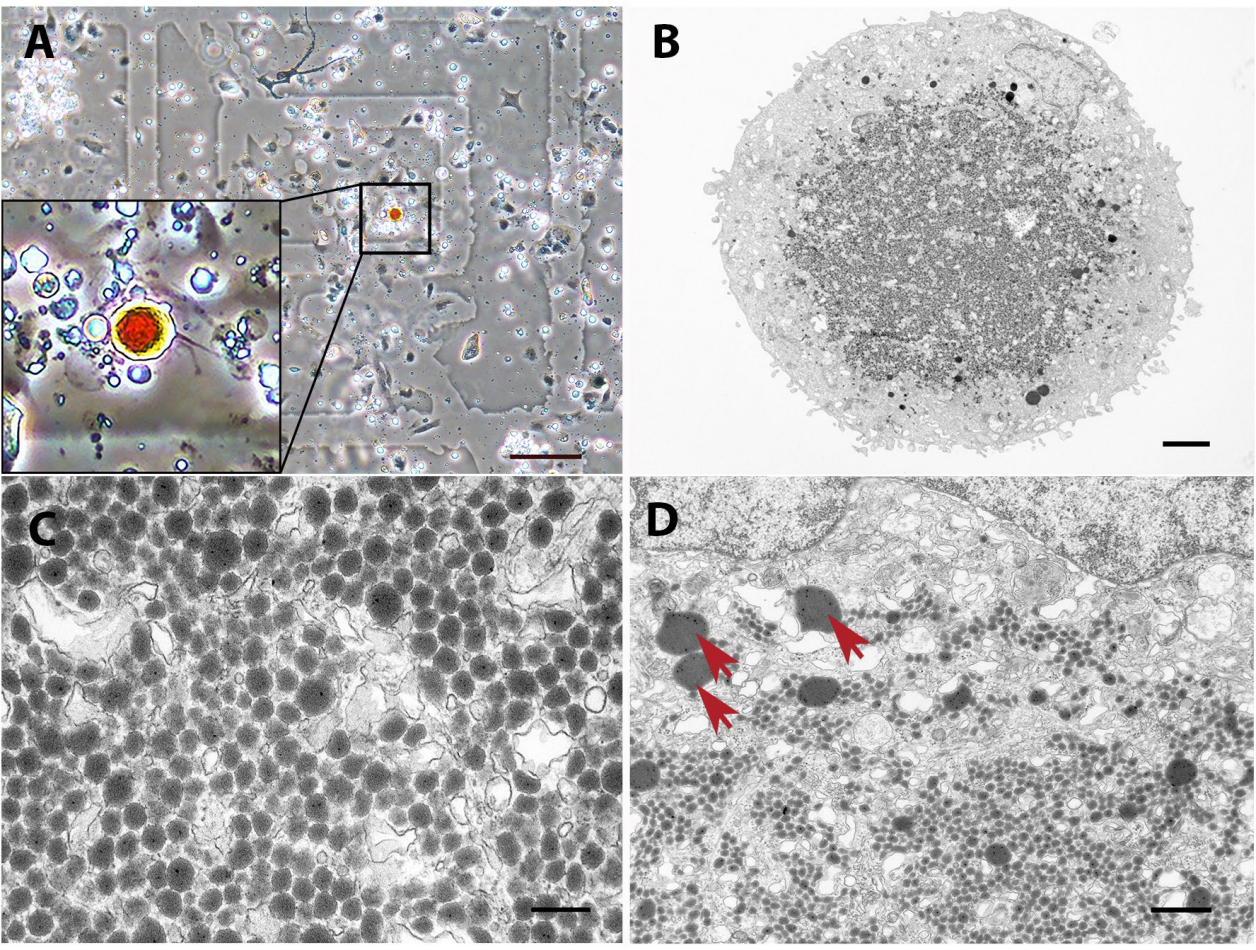
CLEM of a type 1 erythrophore from black spot. (**A**) An isolated type 1 erythrophore on a MatTek dish, observed with phase-contrast microscopy. (**B**–**D**) Ultrastructure of the same cell, observed with TEM. Carotenoid vesicles (red arrowheads), singular erythrosome-like larger inclusions (red arrows). Scale bars: 100 μm (**A**); 2 μm (**B**); 200 nm (**C**); 600 nm (**D**).

**Figure 4:**
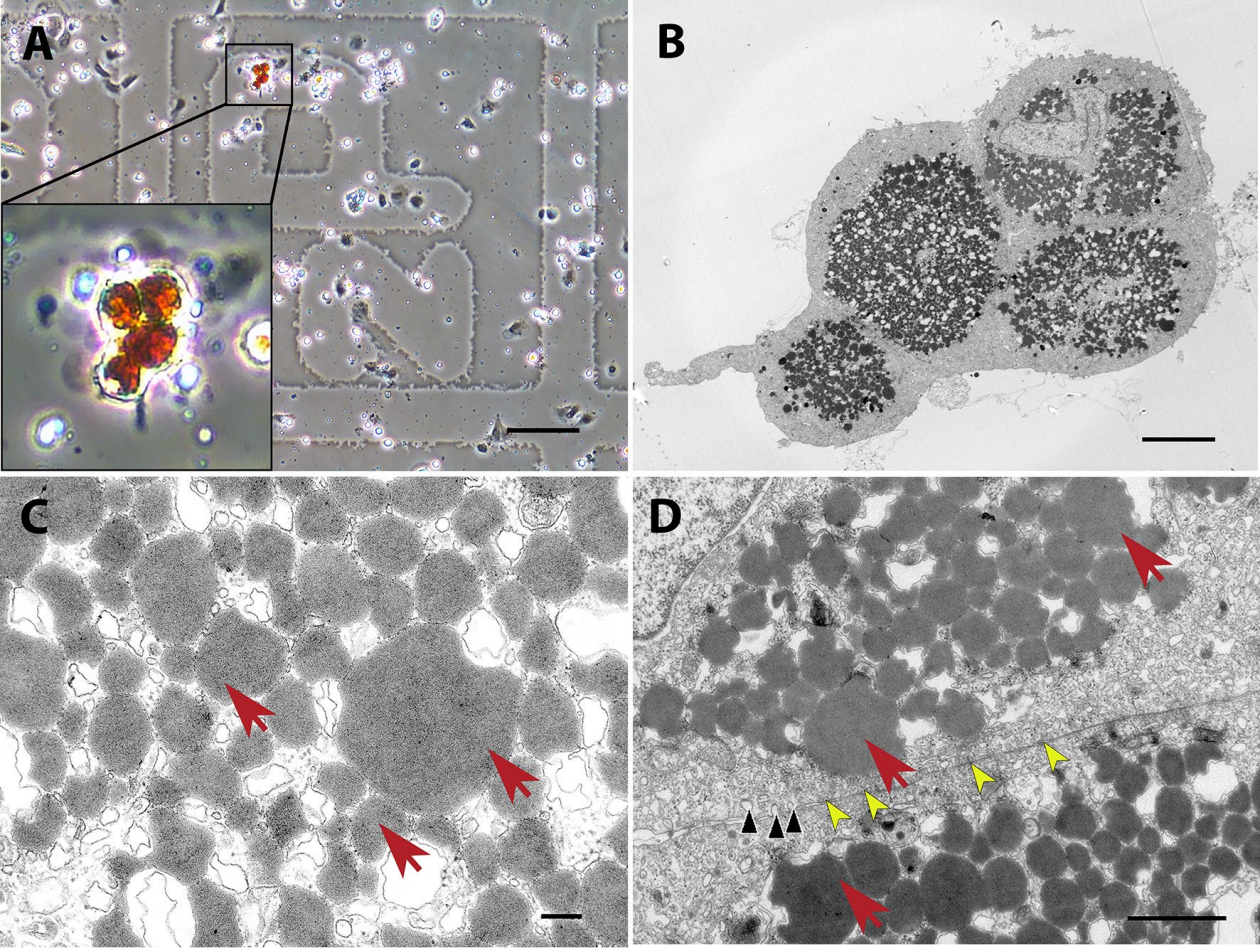
CLEM of a type 2 erythrophore from red spot. (**A**) Four isolated erythrophores on a MatTek dish, observed with phase-contrast microscopy. (**B**–**D**) Ultrastructure of the same cells, observed with TEM. Erythrosomes (red arrows). (**D**) Numerous caveolae near the cell membrane (black arrowheads) and cell junctions (yellow arrowheads). Scale bars: 100 μm (**A**); 6 μm (**B**); 200 nm (**C**); 1 µm (**D**).

Using the CLEM approach and in vitro culturing of cells from the black and red spots of brown trout skin, we confirmed the presence of two erythrophore types/subtypes/states, which differ mainly in size and inclusion type (Figs. 3 and 4). We confirmed the presence of carotenoid vesicles in type 1 erythrophores and erythrosomes in type 2 erythrophores. These inclusions show great homology within a cell type but significant size differences between type 1 and 2 erythrophores. The mean diameter of the carotenoid vesicles in type 1 erythrophores was 93 ± 47 nm (Fig. 3A–C), with some individual vesicles reaching a diameter of 500 nm (Fig. 3D). The diameter of erythrosomes in type 2 erythrophores ranged from 200 nm to 1 μm (412 ± 185 nm) (Fig. 4A–D). We attributed the differences in the measured carotenoid vesicle sizes between in situ and CLEM analyses to the sample size of the CLEM analysis, where only one type 1 erythrophore and four type 2 erythrophoress were observed and measured.

The inclusions in erythrosomes and carotenoid vesicles are darker in CLEM preparation than the samples prepared routinely for TEM, most probably due to the same incubations time in 2% osmium tetroxide and staining with 2% uranyl acetate in sterile distilled water (both 2 h at RT), although the size and thickness of CLEM samples were significantly smaller.

### Gene expression analyses

**Figure 5:**
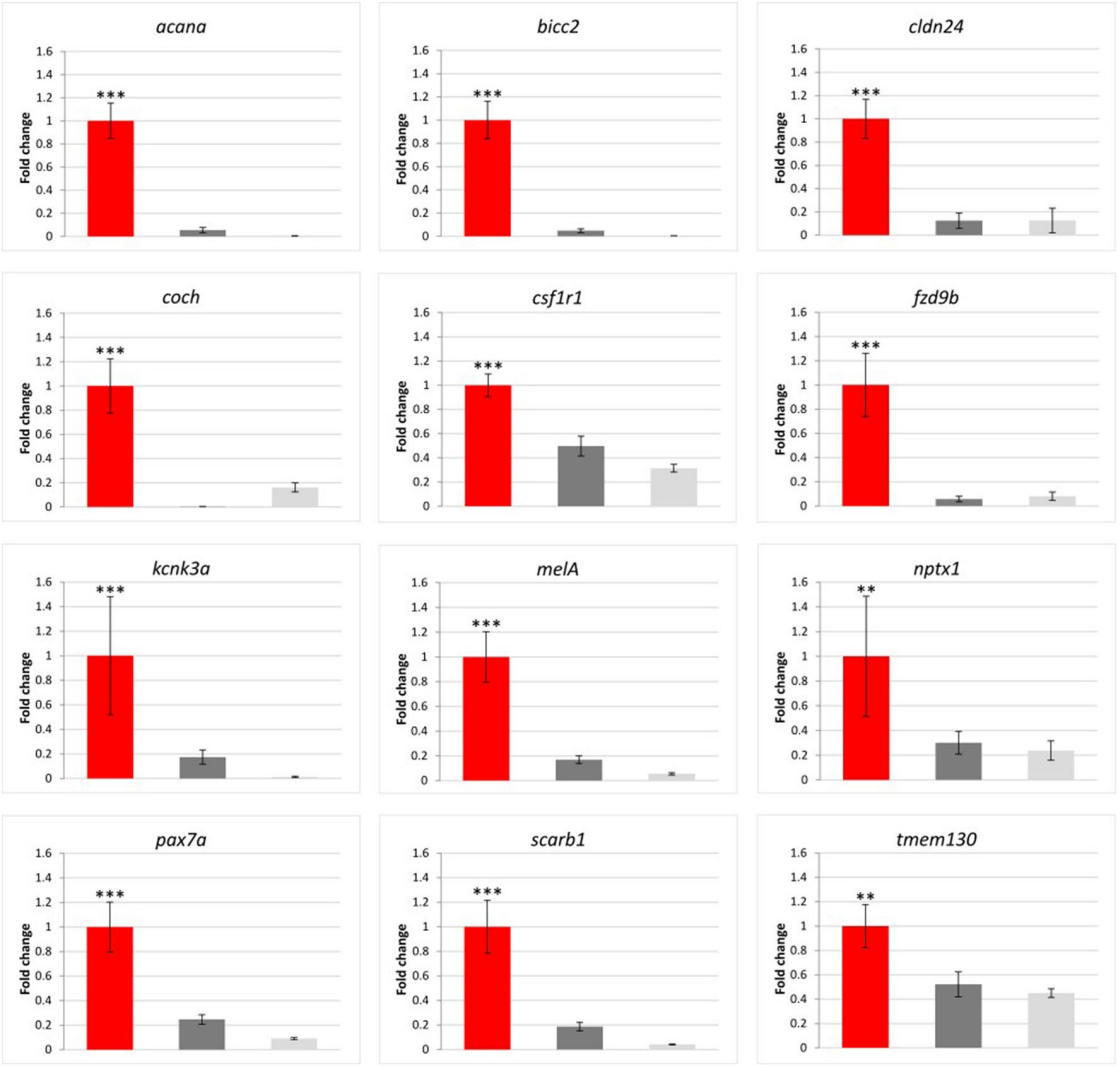
Gene expression patterns obtained with qPCR. Fold changes are expressed as the ratio of gene expression after normalisation to the reference genes. The error bars represent the mean ± SE, and the bars are coloured in red (red spots), dark grey (black spots), and light grey (background colour regions). The expression of each gene was compared with its expression in the red spots and statistically evaluated using the unpaired Student’s t-test. All genes were significantly upregulated in the red spots compared with the other two analysed skin regions. ***p < 0.001; **p < 0.01.

Using qPCR, the expression of 12 genes was analysed in 24 brown trout samples (eight individuals, each with three skin regions: black spots, red spots, and the background colour region). The qPCR results were consistent with the transcriptome sequencing data. All the genes were upregulated in the red spots, compared with the black spots or background colour regions, and followed the same expression trend: red spot > black spot > background colour region (Fig. 5). For ten of these genes (*acana*, *bicc2*, *cldn24*, c*och*, *csf1r1*, *fzd9b*, *kcnk3a*, *melA*, *pax7a*, and *scarb1*), highly significant differential expression was detected in red spots compared with black spots and background colour regions (p < 0.001). Eight of these genes (*acana*, *bicc2*, *cldn24*, *coch*, *fzd9b*, *kcnk3a*, *melA*, and *scarb1*) were upregulated by ≥ five-fold in the red spots.

The enrichment analysis of 16 genes (12 from this study and an additional 4 with the same expression pattern: *mc1r, ednrb, mitf,* and *sox10* from^9^) showed that the only enriched gene ontology (GO) term was transmembrane signalling receptor activity (GO:MF), when brown trout (*S. trutta)* was selected. When analysing orthologues in *D. rerio*, multiple enriched GO terms were connected with pigmentation (GO:BP: pigment cell differentiation, developmental pigmentation, pigmentation, xanthophore differentiation, regulation of developmental pigmentation, and iridophore differentiation; GO:CC: plasma membrane and cell periphery; KEGG pathway: melanogenesis; all p < 0.05).

**Figure 6:**
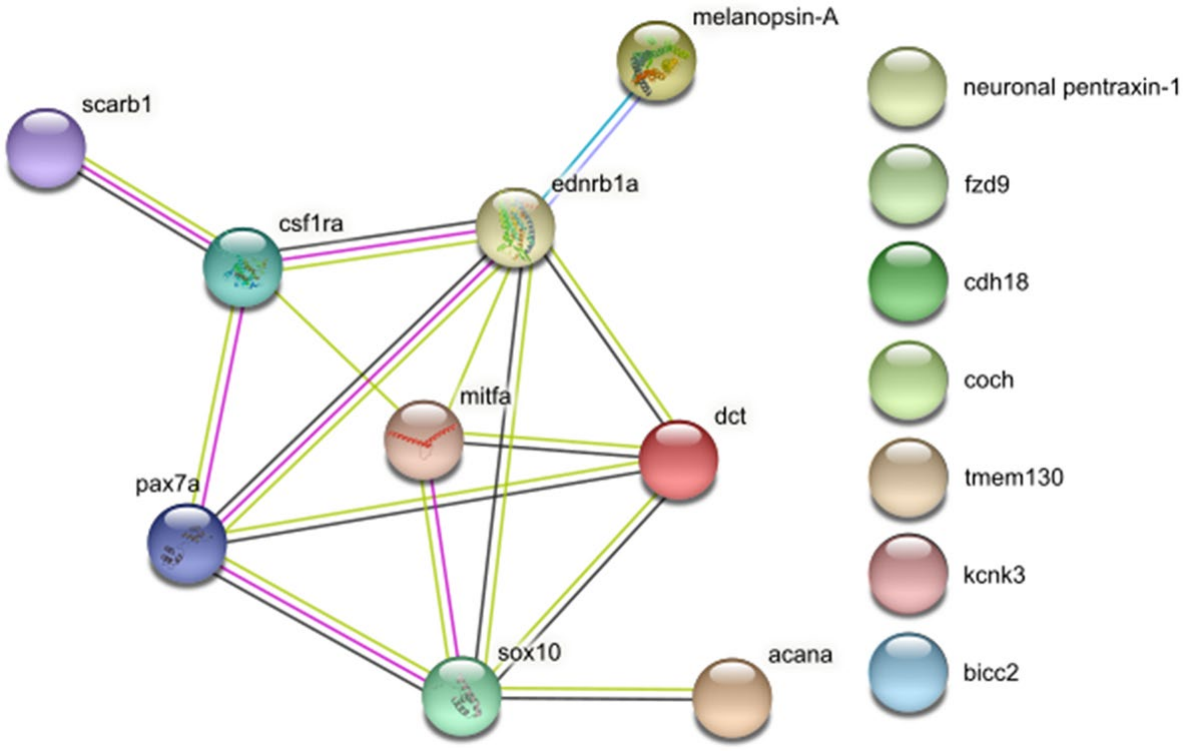
A protein–protein interaction map of zebrafish gene orthologues. The analysis was performed using STRING^10^, claudin-24 was not included. The interactions were determined and colour-coded by homology (violet), co-expression (black), experiments (pink), databases (turquoise), and text mining (light green).

A STRING^10^ analysis was also done on the same 16 candidate genes to determine protein interactions. Putative homologues of these genes in *D. rerio* were analysed, except for *claudin-24*, which is not present in the *D. rerio* genome. The enriched KEGG pathway in the network was melanogenesis, and transmembrane signalling receptor activity as a molecular function. Multiple interactions, including co-expression, are evident among these genes (Fig. 6).

### Paralogue gene analyses

Blast hits of analysed transcripts and their locations are reported together with paralogue gene information (Table 1). Except for *bicc2* and *tmem130*, which do not have paralogues in the brown trout genome, all the other analysed genes have two, three, or even four paralogues in the brown trout genome, stemming from either salmonid-specific fourth vertebrate whole-genome duplication (Ss4R) or both Ss4R and fish-specific genome duplication (FSGD) events (Table 1). Primer blast for most of the analysed loci revealed that transcripts from only one paralogue were quantified with qPCR (original primers perfectly matched both paralogues only for the gene *nptx1*).

**Table 1:** Results of the original primer blasts against the brown trout genome.

Gene name	Ensembl ID	Location	Ss4R paralogues	FSGD paralogues
acana*	ENSSTUG00000018681	29	ENSSTUG00000037301 (12)*	–
*bicc2*	ENSSTUG00000000142	3	–	–
*cldn24*	ENSSTUG00000028911	27	ENSSTUG00000011864 (9)	–
*csf1r1*	ENSSTUG00000006727	3	ENSSTUG00000003249 (13)	–
*coch*	ENSSTUG00000022865	33	–	–
*fzd9b*	ENSSTUG00000041376	13	ENSSTUG00000022387 (26)	ENSSTUG00000023903 (15)
*kcnk3a*	ENSSTUG00000002375	1	ENSSTUG00000043204 (35)	–
*melA (opn4b)*	ENSSTUG00000008119	2	ENSSTUG00000040347 (10)	–
nptx1**	ENSSTUG00000043806	14	ENSSTUG00000021487 (38)**	–
pax7a*	ENSSTUG00000008874	16	ENSSTUG00000004759 (14)*	ENSSTUG00000016476 (28)
*scarb1*	ENSSTUG00000049332	23	ENSSTUG00000023406 (12)	–
*tmem130*	ENSSTUG00000003928	18	–	–
*ednrba*	ENSSTUG00000006342	31	ENSSTUG00000010807 (21)	–
*mitfa*	ENSSTUG00000014914	14	ENSSTUG00000034903 (16)	ENSSTUG00000032663 (28) and ENSSTUG00000037895 (30)
sox10*	ENSSTUG00000036512	32	ENSSTUG00000033003 (1)*	–
*mc1r*	ENSSTUG00000014295	29	ENSSTUG00000020496 (12)	–

The Ensembl ID of the best hit and primary assembly location (considered as chromosome number) are reported. For paralogues, duplication events (Ss4R/FSGD), Ensembl IDs, and locations (in brackets next to Ensembl ID) are reported. Primers that match both paralogues are marked with ** next to the gene name; * denotes that one of the primers has one or two nucleotide mismatches to other paralogues. In both cases, both paralogues could be potentially amplified in the first qPCR analyses.

Three genes were selected for more in-depth analyses: *mitfa*, *sox10,* and *pax7a*. Two copies of the *mitf* gene are known in zebrafish, *mitfa* and *mitfb*, as a result of FSGD. The Ss4R promoted the presence of four paralogues of *mitf* in the brown trout genome (*mitfa* on chromosomes 14 and 16 and *mitfb* on chromosomes 28 and 30; Table 1). Closer analyses of the transcriptome data and transcript blasting of the brown trout genome revealed that transcripts from all four paralogues are expressed in brown trout skin. The paralogue on chromosome 14 was predominantly expressed in red spots, while its expression in other skin regions was considerably lower. The original primers designed for qPCR specifically aligned to this paralogue, and thus only the expression of *mitfa* coded on chromosome 14 was detected with qPCR, which revealed its upregulated expression in red spots compared with other skin regions. To determine the expression profiles of paralogue gene copies in differently pigmented skin regions, paralogue-specific primers were designed for the remaining three paralogues of *mitf* (positioned on chromosomes 16 (*mitfa*), 28, and 30 (both designated as *mitf*) (Table 1)). No differences in the expressions of the other three *mitf* paralogues were detected between differently pigmented skin regions in brown trout. All four *mift* paralogues code for functional proteins with potentially altered transcription activation (see Supplementary information online for more details).

**Figure 7:**
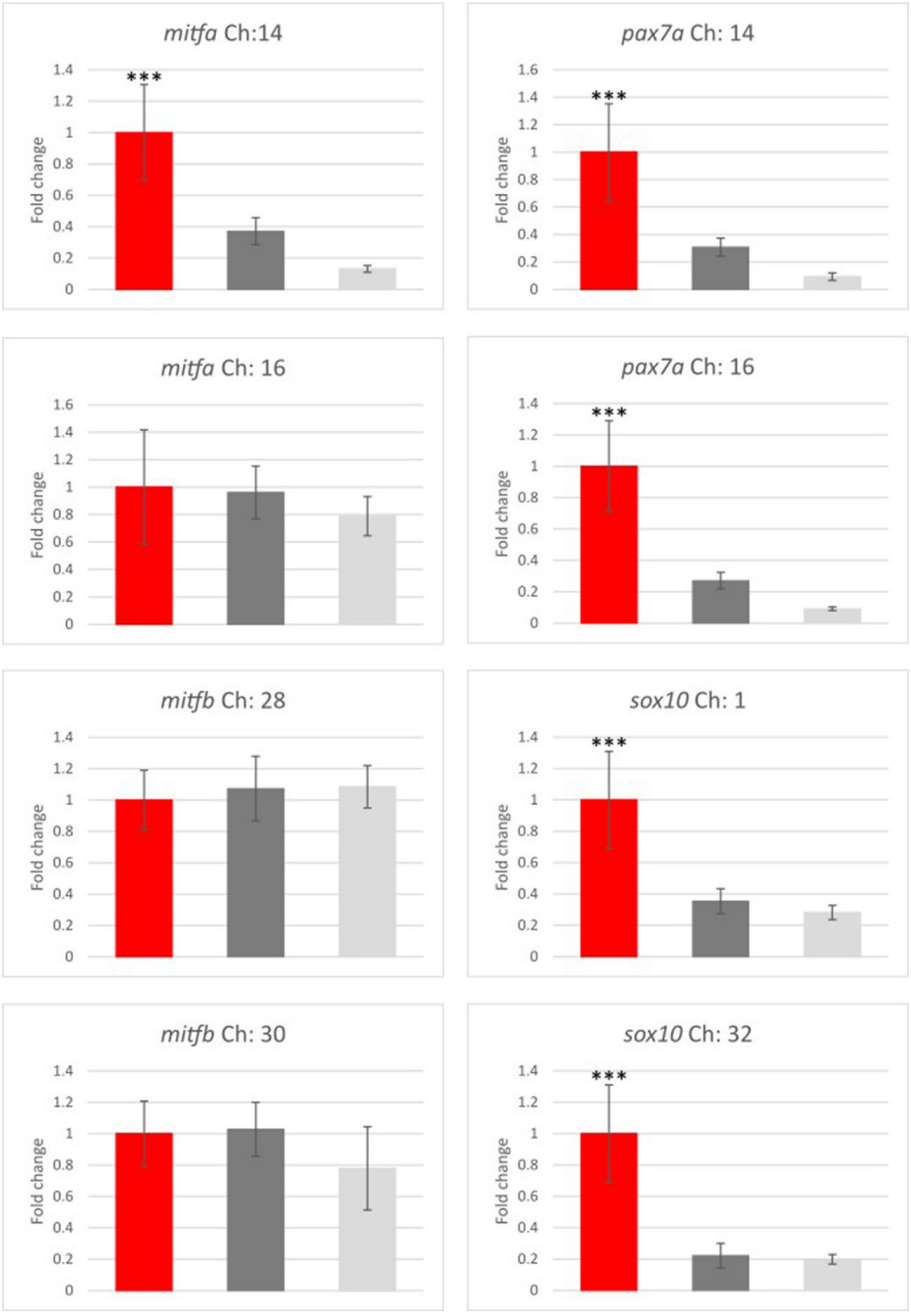
Paralogue gene expression patterns obtained with qPCR. The location of the paralogue gene copy is marked next to the gene name as chromosome number (Ch). Fold changes are expressed as the ratio of gene expression after normalisation to the reference genes. The error bars represent the mean ± SE, and the bars are coloured in red (red spots), dark grey (black spots), and light grey (background colour regions). The expression of each gene was compared with its expression in the red spots and statistically evaluated using the unpaired Student’s t-test. ***p < 0.001.

The brown trout genome contains three paralogues of *pax7* (two copies of *pax7a* stemming from Ss4R and *pax7b*), whereas most teleost fish have two copies (*pax7a* and *pax7b* as a result of FSGD). Two paralogues of *sox10* are also present in the brown trout genome (stemming from Ss4R), whereas no paralogue from FSGD is retained in teleost genomes. The original primers preferentially amplified one of the copies (stemming from Ss4R, Table 1) of both these genes; however, since only one or two nucleotide mismatches in the primer annealing site were present in the other paralogue, it is possible that both paralogues were amplified. New primers were designed to specifically amplify each of the paralogues (*sox10* positioned on chromosomes 1 and 30, in both cases designated as *sox10,* and *pax7a* positioned on chromosomes 14 and 16, in both cases designated as *pax7a*) and to analyse the paralogue expression profiles in differently pigmented skin regions in brown trout (Fig. 7). Both analysed *pax7a* paralogues were upregulated in the red spots compared with the black spots and background colour regions. The lowest expression of both paralogues was detected in the background colour region, in which xanthophores were described as the dominant chromatophore^4^. Similarly, the expression of both *sox10* paralogues was upregulated in the red spots (Fig. 7). Interestingly, none of the analysed paralogues were upregulated in the black spots, in which melanophores predominate, or in the background colour region, in which xanthophores predominate^4^.

## Discussion

Previously, TEM revealed that erythrophores can be divided into two types: type 1 with carotenoid vesicles and type 2 with markedly larger erythrosomes (up to 1 μm in diameter)^4^. In the present study, the differences between the two erythrophore types or states were additionally confirmed using TEM and CLEM, which revealed that the inclusion sizes significantly differ (p < 0.001) between the two cell types. However, the present study has revealed that type 1 erythrophores can contain rare larger inclusions/vesicles (with diameters of up to 1 µm), which closely resemble the erythrosomes found in type 2 erythrophores (which, however, have diameters of up to 3.5 µm), and that type 2 erythrophores contain also some smaller inclusions. These findings blur the boundary regarding cell inclusion size, which was the main criteria for distinguishing between the two different erythrophore types in our first study^4^. Therefore, we here question the validity of defining two types of erythrophores and rather propose describing them as cell subtypes or differentiation states^11^. To this end, the term subtype, instead of type, is hereafter used to differentiate between erythrophores originating from either black or red spots of brown trout skin.

Subtype 2 erythrophores seem to be stabilized and firmly integrated into red spots by fibroblasts or fibroblast-like cells, which were well connected via numerous desmosomes as well with subtype 2 erythrophores. Subtype 2 erythrophores in red spots were better connected with cell junctions than subtype 1 erythrophores in black spots. This was noticed also during sample preparation for CLEM, where four subtype 2 erythrophores have remained connected whereas subtype 1 erythrophores were always solitary. Moreover, in red spots, subtype 2 erythrophores were surrounded by a prominent network of collagen fibres that presumably hinder the migration of erythrophores out of the red spots.

Multiple genes were found to be upregulated in the red spots compared with other skin regions^9^. With a more detailed analyses of our previous results and an additional assessment of differential gene expression using qPCR, the present study showed a unique pattern of gene expression in the red spots of brown trout, which are characterised by subtype 2 erythrophores.

Although we speculate that the analysed genes are chromatophore-specific, it must be emphasized that the expression was analysed using the whole skin sample, including the epidermis and dermis with chromatophores and surrounding cells. Thus, both the type of chromatophore and specific homo- and hetero-typic communication between chromatophores and their surrounding cells might lead to specific expression profiles for particular skin regions.

Among the tested genes upregulated in the red spots, *ednrba*, *mitfa*, *fzd9b,* and *mc1r* are involved in melanogenesis; *pax7a* and *scarb* are involved in xanthophore differentiation and carotene metabolism; and *sox10* is involved in chromatophore differentiation from neural crest cells^12–15^. The lineage relationships between erythrophores (particularly subtype 2) and other cell types in salmonids are unknown. However, our results imply a very specific gene expression profile for erythrophores, with overexpressed genes associated with melanophores and partially xanthophores. Xanthophores have never been found in red spots, whereas individual melanophores are present above the erythrophore layer (^4^ and this study). Interestingly, *mitfa, pax7a,* and *sox10* are essentially involved in the differentiation of pigment cells and can also be expressed in pigment cell precursors/progenitors in zebrafish^12,16,17^. A common chromatophore precursor or stem cell has been postulated^18^ and has been suggested to be located in the dorsal region of adult zebrafish^19^. It was also demonstrated that *sox10* acts cell-autonomously in all pigment cell lineages^20^. Furthermore, it was hypothesised that in zebrafish, melanoblast- and xanthoblast-specific markers, such as *mitfa, csf1r1*, *xdh,* and *pax7,* are co-expressed in a sub-population of dorsal chromatophore precursors^16^. Interestingly, many of the genes that were identified in zebrafish as characteristic of chromatophore precursors were co-expressed and upregulated in erythrophore-containing red spots in brown trout.

Erythrophores have rarely been described in salmonid species^8^ and are poorly characterised. Unlike xanthophores, which are present in all salmonid species and in all developmental stages, erythrophores are not found in all salmonid species nor in all stages of ontogenesis^8,21^. Interestingly, a similar situation was found in the *Danio* clade, for which the phylogenetic distribution of species with erythrophores suggests that this cell type may have independently appeared and disappeared multiple times^22–24^. Most adult *Danio* species have either xanthophores or erythrophores on their bodies^23^. Recently, Huang et al.^24^ described adult skin pigment pattern of *D. albolineatus*, where both cell types are present, but in distinct skin regions (bands) on body and fins, where they are the only pigment cell type present. Similarly, both cell types are present simultaneously also in brown trout, although they do never appear together (no direct cell–cell interactions exist between them^4^). While xanthophores are distributed across almost all skin regions in salmonids, erythrophores are mainly distributed within spots or lateral lines^4,21^.

Subtype 2 erythrophores are of special interest, since they are the predominant cell type in red spots, characterised by a particular cell ultrastructure and gene expression profile. However, our analyses could not determine the gene expression profile of subtype 1 erythrophores or xanthophores. These last two cell types are skin-region specific; however, they rarely undergo homotypic interactions but rather undergo heterotypic interactions with other cells (^4^ and this study). Some of the analysed genes were overexpressed also in black spots, in which subtype 1 erythrophores are present in a small proportion, compared with background colour regions. Together with two additional genes with almost identical expression patterns between black and red spots (*gja5* and *dct*^9^), the gene analysis in the present study suggests at least partially similar expression patterns in both erythrophore subtypes. Nevertheless, the distinct expression profiles with highly upregulated genes in the red spots indicate a regulatory independence^25^ of subtype 2 erythrophores.

It has been shown already that pigment cells in zebrafish can differentiate into different cell types or states, based on their micro-environment^5^. Iridophores obtained a particular morphology (organization of guanine-reflecting platelets) in zebrafish stripes and interstripes during their differentiation that depended on the presence or absence of melanophores. The iridophores of interstripes and stripes also exhibited distinct transcriptomic states, indicating that they represent different cell types^5^. A similar model of cell differentiation could also be proposed for erythrophores in brown trout. We suppose that cell communication with melanophores and particular paracrine signalling might promote differentiation into subtype 1 erythrophores in black spots, while homotypic cell communication with paracrine/autocrine signalling promotes differentiation into subtype 2 erythrophores in red spots.

The role of the tissue microenvironment in pattern formation and maintenance has been highlighted multiple times^26–28^. To date, many studies have described the diverse mechanisms of cell communication between fish chromatophores and surrounding cells (and their influence on chromatophore distribution and pigment patterns), including gap junctions, potassium channels, long-range communication through cellular extensions (and Delta/Notch signalling), macrophage-mediated communication through airinemes, pseudopodia communication, and communication via diffusing signals (reviewed in^27^). Although many genes have been characterised as specific to subtype 2 erythrophores, these genes that are upregulated in the red spots of brown trout could also be differentially expressed in the cells surrounding the erythrophores, and this could influence chromatophore distribution and pigment patterning via yet unknown mechanisms of communication. Surrounding cells may also directly affect the differentiation state of the two erythrophore subtypes and subsequently their gene expression. For example, melanophores may affect subtype 1 erythrophores, which may consequently never reach the final cell differentiation state that is presumably present in red spots.

The key to the origin of a new cell type, subtype, or state is the regulatory independence of a cell; that is, the ability of a cell to regulate and evolve gene expression independently of other cells^25,29,30^. The process of gene duplication is believed to be a major factor contributing to the generation of novel gene functions within a genome^31,32^. Following genome duplication, the functions of duplicated genes can either be retained, lost, or can diverge. In teleost fish, pigmentation genes have been preferentially retained in duplicate after FSGD, so that teleost fish have 30% more pigmentation genes than tetrapods^1,33^. It has been shown that large parts of the melanophore and xanthophore pathways are present in two copies in teleost fish^2,33^. It has also been concluded that FSGD has made an important contribution to the evolution of teleost-specific features of pigmentation, which include novel pigment cell types or the division of existing pigment cell types into distinct subtypes^33^. Furthermore, salmonids experienced another whole-genome duplication (Ss4R) about 80 million years ago^34^, resulting in multiple paralogues of pigmentation genes in their current genome. Given the above and based on our results, we suggest that the duplication events FSGD and/or Ss4R may have also promoted the emergence of new cell subtypes in brown trout when duplicated genes or regulatory regions underwent neofunctionalization. Although, subfunctionalization could also be considered as regulatory fate for gene duplicates, neofunctionalization was suggested as predominant evolutionary fate of genes after the whole genome duplication events in salmonids^35^. Detailed analyses of *mitf* paralogue gene and protein copies (please see Supplementary Figs. S1–S4 and Table S1 online) are also supporting the hypothesis of duplicated gene neofunctionalizations.

As already mentioned, lineage relationships between erythrophores (regardless of their type, subtype, or differentiation state) and other cell types in salmonids are unknown. However, as we demonstrated, many differentially expressed genes in subtype 2 erythrophores (or in red spots) are involved in melanogenesis, which is characteristic of melanophores, and in xanthophore differentiation. Our detailed analysis of these genes and their transcripts revealed that two or even four paralogues are present in the brown trout genome; however, the expression of only one of them was analysed at first. Paralogues in salmonids are the consequence of two genome duplication events (FSGD and Ss4R). Thus, it is possible that erythrophores (subtype 2 or both subtypes) developed as a consequence of duplicated gene neofunctionalization, either from melanophores, xanthophores, or their common precursors/progenitors derived from neural crest cells. Differentiation into erythrophores might be further triggered by paracrine or autocrine signalling, promoting the expression of duplicated gene copies. Recently, Huang et al.^24^ showed that erythrophores in the fin of *Danio albolineatus* share a common progenitor with xanthophores and maintain plasticity in cell fate even after differentiation.

Nevertheless, similarities between the functions of melanophores and xanthophores in metabolic pathways and during development have also been demonstrated^36,37^. It was even proposed that functional melanin and coloured pteridine pathways are present in both melanophores and xanthophores, and that this could enable chromatophore transdifferentiation (i.e. the transformation of one type into another)^8,38^. Furthermore, mosaic pigment cells containing more than one type of organelle, and mosaic organelles containing more than one type of pigment can be found in vertebrate skin^8,39^. For instance, in frogs, a mosaic of chromatophores containing melanosomes, erythrosomes, xanthosomes, and refractosomes was observed^40,41^. This suggests evolutionary relationships between these cells and possible transdifferentiation of already differentiated chromatophores^8^. In vitro and interspecies transplantation experiments demonstrated that amphibian xanthophores and iridophores can transform into functional melanophores and vice versa^38,42^. These findings highlight the proximity of the melanin and pteridine pathways, suggesting that, in teleost fish, established melanophores may also differentiate into xanthophores or erythrophores, or that xanthophores can differentiate into erythrophores, and vice versa. The differentiation of xanthophores into erythrophores would require even fewer changes in the function of genes and metabolism in addition to requirements for several genes in red or yellow coloration, as recently demonstrated in *D. albolineatus*^24^. Similarly, studies on medaka (*Oryzias latipes*) showed that a changed mode of interaction between only two genes (*Sox5* and *Sox10*) affects the specification of pigment cell types and, by affecting the xanthophore progenitor, correlates with the evolution of a novel leucophore pigment cell type^43^. Differential expression of one or more paralogues might thus promote the differentiation of chromatophore precursors to a novel pigment cell subtype or state, e.g. erythrophores in brown trout.

The expression profile of paralogue genes is shown to be only partially differentiated in differently pigmented brown trout skin regions. However, there is no clear distinction between the expressions of individual paralogues in differently pigmented skin regions, with the exception that multiple paralogues are overexpressed in red spots compared with other skin regions. These results still suggest that duplicate gene copies at least partially adopt new functions (e.g. *mitfa* coded on Ch:14, more data about *mitf* nucleotide and amino acid alignments and protein structure is presented in Supplementary information online) and contribute to the transformation of a new chromatophore subtype that is involved in red spot formation. It is hypothesised that erythrophores and/or surrounding cells in red spots partially preserve the expression profile of the precursor/progenitor pigment cell (i.e. *sox10* expression, related to the findings of^43^ mentioned above) or that chromatophore precursors are present in red spots, generating subtype 2 erythrophores. An additional change in the expression of one of the duplicated gene copies might trigger the emergence of a new cell subtype (e.g. an erythrophore). Nevertheless, the surrounding cells, which were analysed together with chromatophores in differently pigmented skin regions, probably also play an important role in chromatophore positioning and pigment patterning. The specific expression profile and interactions of the surrounding cells may also contribute greatly to the transformation/differentiation state of a cell type or the emergence of new cell type/subtype.

In addition to the new hypothesis regarding cell subtype origin, chromatophore transdifferentiation as a mechanism of subtype 2 erythrophore origin in brown trout skin should also be considered. Transdifferentiation of chromatophores in amphibians has already been discussed above. An example of pigment cell transdifferentiation was also demonstrated in zebrafish^44^. Two classes of leucophores are characteristic of zebrafish skin, and one of them, melanoleucophores, develop directly from melanophores, accumulating white material and losing melanin. Using transcriptomes analyses of individual pigment cells during melanoleucophore development, the authors demonstrated that the differentiation of melanoleucophores from melanophores involves a switch from melanin to purine synthesis^44^.

The present study has opened a number of hypotheses regarding erythrophore origin and differentiation into two types, subtypes, or states in brown trout and has also provided an important basis for further analyses. Defining cell types and states and tracing their developmental origin requires single-cell assays^11^. Therefore, single-cell sequencing of all chromatophore types and subtypes, including other cells surrounding the chromatophores, needs to be performed in brown trout. Although single-cell approaches are revolutionizing our understanding of cell structure and gene regulation, it has to be noted that some information is lost when focusing on a single cell, as revealed also in this study using CLEM and in situ cell observations. Furthermore, observing pigment cell emergence and pattern formation during all life stages as well as cell (trans)differentiation in vitro and its dependence on interactions with other cells (i.e. subtype 2 erythrophores with melanophores) could be a promising approach to determine the appropriateness of classifying cells into types or subtypes.

## Materials and methods

### Material

**Figure 8:**
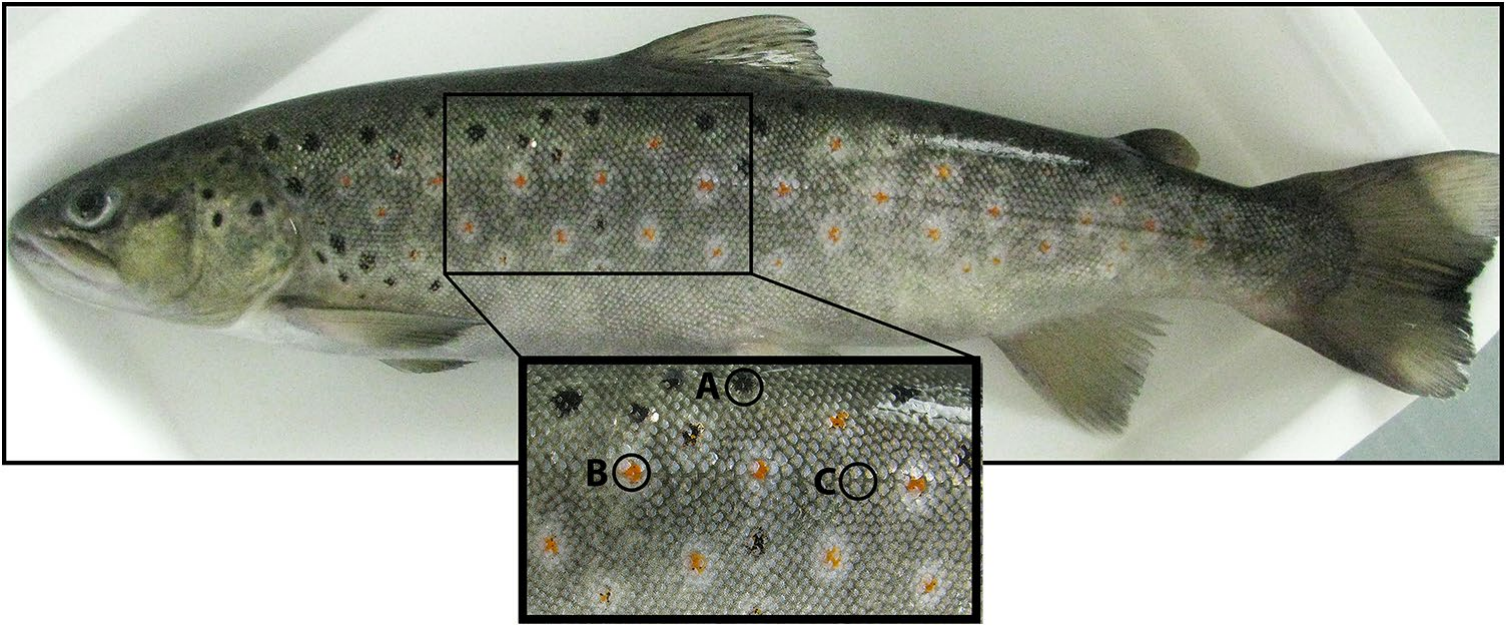
The analysed skin regions of brown trout. (**A**) black spot, (**B**) red spot, and (**C**) background colour region.

Ten adult brown trout individuals (aged > 2 years) were collected from Bled fish farm, Bled, Slovenia. Their average body length was approximately 30 cm. All fish were first-generation offspring reared in the fish farm from wild-caught parents originating from Malešnica stream, Slovenia. Individuals had been fed with Biomar INICIO Plus (Denmark) as a starter feed and Biomar EFICO Enviro 920 during adulthood. Prior to skin sample collection, the fish were sedated in anaesthetic Tricaine-S (MS-222, Western chemical, Ferndale, USA) and killed by a blow to the neck. Small pieces of skin from the lateral part of the trunk of the body (Fig. 8) were obtained with a 2 mm biopsy punch (Kai Group, Japan) that selectively dissected differently pigmented skin regions (background colour region and black and red spots). These pieces of skin were used for CLEM and gene expression analyses as described below. All methods described were carried out in accordance with relevant guidelines and regulations. Fish skin sampling was approved by the Ministry of Agriculture and Environment, Slovenia, under decision letter number U34401–60/2013/4. The study was carried out in compliance with the ARRIVE guidelines^45^.

### Light microscopy and TEM

The skin samples of two brown trout were dissected and observed directly under a light microscope (Nikon Eclipse, TE-2000U). Small pieces of skin from the lateral part of the trunk of the body (Fig. 8) were obtained with a 2 mm biopsy punch (Kai Group, Japan) to selectively dissect differently pigmented skin regions (i.e. the black and red spots). The skin samples were immersed in a mixture of 3% paraformaldehyde and 3% glutaraldehyde in 0.1 M cacodylate buffer, fixed overnight at 4 °C, and subsequently fixed in 2% osmium tetroxide in sterile distilled water for 2 h at room temperature. Samples were then processed as described previously^4^. Briefly, the samples of two red and two black spots were stained *en bloc* with 2% uranyl acetate in sterile distilled water for 2 h, dehydrated in a graded series of ethanol solutions, and embedded in Epon 812 resin (Serva Electrophoresis, Heidelberg, Germany). Semi-thin (1 µm) and ultra-thin (65 nm) sections were cut using an Ultracut UCT microtome (Leica, Austria). Semi-thin sections were contrasted with toluidine blue and examined under a light microscope to determine the position of the pigment cells. The centres of the black and red spots were cut into ultra-thin sections, which were then contrasted with 2.5% of filtered uranyl acetate in sterile distilled water and lead citrate solution prepared according to the protocol described in^46^ and examined under a CM100 transmission electron microscope (Philips, Eindhoven, Netherlands; operation voltage 80 kV) equipped with a CCD camera (AMT, Danvers, MA, USA) for cell ultrastructure analysis. Altogether we analysed two independent red and two independent black spots, from which we altogether took 49 images. Twenty-seven images were taken for red spot, 16 and 11 from each red spot sample, respectively. Similarly, 22 images were taken for black spot, 16 and 6 from each black spot, respectively.

### CLEM of the cells in the black and red spots

CLEM combines the strengths of light microscopy and electron microscopy, enabling complementary analysis of cellular events or structures within the same sample. This method was used to further describe the two erythrophore types in brown trout skin.

Pigment cells were isolated separately from the red spots that mostly contain type 2 erythrophores and from the black spots that mostly contain melanophores and type 1 erythrophores. We followed the protocol described in^47^ with slight modifications. Briefly, pieces of skin were treated with trypsin solution (2.5 mg/mL trypsin, 1.2 mg/mL bovine serum albumin, 1 mM EDTA (all Sigma-Aldrich, USA) in phosphate-buffered saline (PBS)) for 80 min at 20 °C. After washing three times with PBS, the samples were treated with a collagenase solution (1 mg/mL collagenase type I (Sigma-Aldrich, USA), 0.1 mg/mL DNase I (Thermo Fisher Scientific, MA, USA), 100 μL/mL foetal bovine serum (Merck, USA), 1.2 mg/mL bovine serum albumin in PBS) for 80 min at the agitation speed of 1000 rpm (Thermal Shake lite, VWR International, USA) at room temperature. The cell suspension was then filtered through a 25 μm pore size mesh and centrifuged at 40 g at room temperature. To separate the pigment cells from other cell types, the cell suspension was further centrifuged over a Percoll gradient (Sigma-Aldrich, USA). The cell pellet was then resuspended in medium (L-15, 5% antibiotic-antifungal; Sigma-Aldrich, USA) and plated on a MatTek Petri dish (MatTek Corporation, Ashland, USA) with a gridded glass bottom coated with Geltrex substrate (Thermo Fisher Scientific). This grid helps locate the exact same cell, which was primarily observed under a light microscope, under an electron microscope. The isolated cells from the black and red spots were cultured separately under ambient light (fluorescent neon lamp) at 20 °C overnight. Only the red-coloured cells from the black and red spots (i.e. from both MatTek Petri dishes) were selected for further analyses. The cells were first observed under a light microscope, after which they were immediately submerged in a mixture of 3% paraformaldehyde and 3% glutaraldehyde in 0.1 M cacodylate buffer fixative overnight at 4 °C. Following immersion in 2% osmium tetroxide in sterile double distilled water for 2 h at room temperature, they were stained with 2% uranyl acetate in sterile double distilled water for 2 h, dehydrated in an increasing gradient series of ethanol solutions, and finally embedded in Epon 812 resin (Serva Electrophoresis, Heidelberg, Germany). Semi-thin (1 μm) and ultra-thin (65 nm) sections were prepared using an Ultracut UCT microtome (Leica, Austria). The semi-thin sections were contrasted with toluidine blue and examined under a light microscope to find the position of a specific pigment cell. Then the regions appropriate for ultra-thin sections were determined. To enhance the contrast of ultra-thin sections, 2.5% of filtered uranyl acetate in sterile distilled water and lead citrate solution prepared according to the protocol described in^46^ were used. The ultrastructure of specific cells was examined with a CM100 transmission electron microscope (Philips, Eindhoven, Netherlands; operation voltage 80 kV) equipped with a CCD camera (AMT, Danvers, MA, USA).

### Gene expression analyses

Small pieces of differentially pigmented skin (i.e. black and red spots and background colour regions) were obtained with biopsy punches and immediately immersed in liquid nitrogen. The pieces of skin were then homogenised with Percellys 24 homogenizer (Bertin corp.) using zirconium oxide beads (Bertin corp). Total RNA was isolated using the RNeasy Plus Universal Mini Kit according to manufacturer’s instructions and subsequently treated with RNase-free DNase I (Thermo Fisher Scientific). Only RNA with good purity, i.e. with A260/280 values of ~ 2.0, checked with a NanoVue spectrophotometer (GE Healthcare, Little Chalfont, Buckinghamshire, UK), was used for cDNA synthesis, which was performed with the High-Capacity cDNA Reverse Transcription Kit (Thermo Fisher Scientific). Twelve DEGs determined with transcriptome analyses (SRP157513^9^) were selected. We used the following selection criteria: i) genes are upregulated (≥ six-fold) in red spots compared with other skin regions, ii) genes are classified into gene groups with a known role in pigmentation (e.g. *fzd9b*, *csf1r1*, *mitf*, and *mc1r*), iii) genes are involved in cell–cell communication or ion channels (e.g. *kcnk3s* and *cldn24*), or iv) genes are involved in extracellular matrix organization (e.g. *acana* and *coch*). According to our previous results^9^, genes in these groups may contribute to cell–cell communication between chromatophores or with surrounding cells and to the migration and positioning of the cells within tissues. qPCR was performed using SYBR Green PCR Master Mix (Thermo Fisher Scientific, MA, USA) and a Viia7 Real-Time PCR System (Applied Biosystem, Thermo Fisher Scientific). All primers for the selected candidate genes (Table 1) were designed based on transcriptome sequences (SRP157513) obtained using Primer-BLAST^48^ with an amplicon size of 90–300 bp and a melting temperature of 60 °C. To check the primer specificity for the gene of interest, all the selected primers were blasted towards sequences in the NCBI database of the selected organisms (Salmonidae or Salmo). Two internal reference genes previously determined to exhibit stable expression in brown trout skin (see^9^) were used: *rps20* and *pgk1*. The expression of the candidate genes was analysed with eight biological replicates, each performed in triplicate. A no-template-control was used to check for potential external contamination. Standard curve analysis was performed for the reference and target genes to assess amplification efficiency, which was comparable between genes. The conditions for all reactions were 50 °C for 2 min, 95 °C for 10 min, followed by 40 cycles of 95 °C for 15 s and 60 °C for 1 min. At the end of each run, a melting curve analysis was performed to confirm a unique amplicon reaction. The differential gene expression results were calculated using the Pfaffl method^49^, normalised to the geometric mean of the above-mentioned reference genes and with the use of amplicon-specific efficiency. The Student’s t-test was performed to assess the statistical significance of the differential expression between samples, and results with p < 0.05 were considered statistically significant.

gProfiler^50^ was used to conduct enrichment analysis for the tested DEGs. The Search Tool for the Retrieval of Interacting Genes (STRING) database version 11.0 (https://string‑db.org; accessed September 2020)^10^ was used to validate and examine the network of associations among all the genes confirmed to be upregulated in the red spots. Since the STRING database does not include data for brown trout or any other Salmonidae taxon, putative homologues of candidate genes in *D. rerio* were analysed.

### Paralogue gene analyses

Transcripts were obtained from transcriptome data, available as PRJNA485234, and blasted against brown trout genome assembly (fSalTru1.1, GCA_901001165.1) using ENSEMBL (Ensembl Release 102, December 2020). The original primers, designed based on transcript sequences, were also blasted against brown trout genome assembly. The top hits were recorded, as well as the paralogue gene copies in the brown trout genome stemming from two duplication events (Ss4R and FSGD).

Paralogue-specific primers were designed based on sequences from the brown trout genome assembly using Primer-BLAST^48^ with an amplicon size of 90–300 bp and a melting temperature of 60 °C (Table 2). Sequences of paralogues (transcripts) were first aligned using MEGA X^51^, and the primers were designed to overlap regions where sequences differ (multiple single nucleotide polymorphisms (SNPs) or indels) to enable paralogue-specific amplification.

**Table 2:** The primers used for qPCR. *Rps20* and *pgk1* are housekeeping genes.

Gene symbol	Ensembl ID	Gene description	Forward primer	Reverse primer
*rps20*	ENSSTUG00000022628	40S ribosomal protein S20	AGCCGCAACGTCAAGTCT	GTCTTGGTGGGCATACGG
*pgk1*	ENSSTUG00000005810	Phosphoglycerate kinase	CTCGGTGATGGGGCTTAGG	TCATTGGTGGAGGCGACA
*acana*	ENSSTUG00000018681	Agrecan core protein-like	TACGAGGCCGGCTACCAC	TGAGCCAGAGCACCGTCATAG
*bicc2*	ENSSTUG00000000142	Protein bicaudal C homologue 1-like	GAGGAATCCGGTGGTGACAG	CGTGGCGTAGCTCCTTAACA
*cldn24*	ENSSTUG00000028911	Putative claudin-24	AACCTGCCTGATGTCGTTCC	TTCTCCTGAGGGGTGTTTGG
*coch*	ENSSTUG00000022865	Cochlin	CACCCATCACCTGTACGACC	GCGTACACCTGAGAACCGAA
*csf1r1*	ENSSTUG00000006727	Macrophage colony-stimulating factor 1 receptor 1-like	GTAATTTTGATGATTGCAGATGCG	GGTGGTGCTGGAGATGATGAATA
*fzd9b*	ENSSTUG00000041376	Frizzled-9-like	CGGCAGGGGCTTTGTAATGA	ACTGTGGCGGTATTCATGCT
*kcnk3a*	ENSSTUG00000002375	potassium two pore domain channel subfamily K member 3	CACCGTCTGAAGAAGTGCCT	CACATAATCCCCGAAGCCGA
*melA*	ENSSTUG00000008119	Melanopsin-A-like, opn4b	ATAACACACCCCAAATACAGGCTA	GCCTCCGTGTCCGTAAATGC
*nptx1*	ENSSTUG00000043806	Neuronal pentraxin-1-like	GCGGCTTGATGGGATGGTAT	GTGGGGGAATAACCCGATGG
*pax7a*	ENSSTUG00000008874	Paired box protein Pax-7-like	ACTGTTCCTTCAGGTGAGGC	CTCCGACTCCACGTCTGAAC
*scarb1*	ENSSTUG00000049332	Scavenger receptor class B member 1-like	GTGGAGCAGAGAGGTCCCTA	TCATCAGACGCACTGGGAAC
*tmem130*	ENSSTUG00000003928	Transmembrane protein 130-like	ACGCTTTTATCCAGAGCCACG	GAGAAATGGGGAGTCTTCAGC
**Paralogues**				
*mitf*a Ch:14	ENSSTUG00000014914	Microphthalmia-associated transcription factor-like	TGAGGACAGAAGGGGGTCAT	TGCGATTCTGCCATCGTCTT
*mitf*a Ch:16	ENSSTUG00000034903	Microphthalmia-associated transcription factor-like	AACCACGGACTTACCAGCAG	TTTGTCTCTCCTTGGCCATC
*mitf*b Ch:28	ENSSTUG00000032663	Microphthalmia-associated transcription factor-like	ATACGATCGCTGTCAACACT	GATCCAGACTTGTCCAGCATA
*mitf*b Ch:30	ENSSTUG00000037895	Microphthalmia-associated transcription factor-like	CCACAATCACACGCACGG	TGGTACTTGGTGGGGTTCTC
*sox10 Ch:1*	ENSSTUG00000033003	Transcription factor Sox-10-like	GGAGCCCTTTGATGTGAACG	CGTAGGCATAGGGAGAAACAG
*sox10 Ch:32*	ENSSTUG00000036512	Transcription factor Sox-10-like	GGAGCCCTTTGATGTGAACG	CGTAGGAATAGGGAGATGCAG
*pax7a Ch:14*	ENSSTUG00000004759	Paired box protein Pax-7	CCTTCAGGTGAGGCTTCATC	CTCCGACTCCACGTCTGAAC
*pax7a Ch:16*	ENSSTUG00000008874	Paired box protein Pax-7-like	CCTTCAGGTGAGGCTTCTGT	CTCCGACTCCACGTCTGAAC

The original primers are listed in alphabetical order according to the gene name. The second half of the table lists the primers for specific amplification of paralogue gene copies.

### Ethics declarations

The study was conducted according to the guidelines of the Animal Protection Act (UL RS, No. 43/07), and approved by the Ethics Committee of Ministry of Agriculture and Environment, Slovenia, under decision letter number U34401–60/2013/4.

## Supplementary Information

Supplementary Information.

## Data Availability

The data presented in this study are available within the article text, figures and in the SRA repository https://www.ncbi.nlm.nih.gov/sra/?term=SRP157513.
