# Supplementary material for: Genetic and correlative light and electron microscopy evidence for the unique differentiation pathway of erythrophores in brown trout skin
Source: Sci Rep. 2022 Jan 19;12:1015. doi: 10.1038/s41598-022-04799-7 (PMC8770521; doi:10.1038/s41598-022-04799-7)
Supplement: Supplementary file 1 — Supplementary Information. [file 41598_2022_4799_MOESM1_ESM.pdf]

SUPPLEMENTARY INFORMATION

Genetic and correlative light and electron microscopy evidence for the unique differentiation pathway of erythrophores in brown trout skin.

Simona Sušnik Bajec[1*], Ida Djurdjevič[1], Carmen Linares Andújar[2], Mateja Erdani Kreft[3]

[1] University of Ljubljana, Biotechnical Faculty, Department of Animal Science, Groblje 3, SI-1230 Domžale, Slovenia

[2] Centro de Investigaciones Biológicas Margarita Salas - CSIC, Calle Ramiro de Maeztu, 9, 28040 Madrid, Spain

[3] University of Ljubljana, Faculty of Medicine, Institute of Cell Biology, Vrazov trg 2, SI-1000 Ljubljana, Slovenia

*Corresponding author: S. Sušnik Bajec

e-mail: simona.susnik@bf.uni-lj.si

tel: + 386 1 320 3944

Representative example comparing paralogue gene copies in brown trout (*S. trutta*): *mitf*

1. Nucleotide and amino acid sequence alignment

Paralogue gene copies of *mitf* in brown trout were compared at nucleotide sequence (cds, coding sequences; Supplementary Figure S1) and protein level (Supplementary Figure S2). Alignments were performed using EMBL-EBI sequence analysis tools [2]: Muscle for nucleotide and Clustal Omega tool for protein alignment were used. Divergence between paralogues is notable throughout the coding sequence with first two and last exon being characterised with multiple nucleotide sequence changes. Examination of the intron-exon structure also shows difference between paralogues, with paralogue on Ch:14 as the most divergent. MiT/TFE transcription factors C-terminal domain stretching over last three exons at paralogue Ch:14, while in other paralogue this domain is bound to the one (last) exon.

The most conserved domains of mitf protein (basic, helix-loop-helix and Leucine zipper) are conserved in all brown trout paralogues, with some conservative substitutions detected (Supplementary Figure S2). According to [3], these changed do not affect the protein function. Hallsson et al.[3] described other conserved region in *mitf* gene, among which Q rich domain and conserved domain 3 in C-terminus with MiT/TFE transcription factors domains are least conserved in brown trout paralogues and could potentially affect transcription activation by mitf.

```
30:MITF-201       ATGCAAGCGGAATCGGGTATAGTTCCTGACTTCGAGGTTGGAGAAGAGTTTCAAGAGGAA
28:MITF-201       ATGCAAGCGGAATCGGGTATAGTTCCCGACTTCGAGGTTGGAGAAGAGTTTCAAGAGGAA
16:mitfa-201      --------------------------------------TGG-------------------
14:mitfa-201      ATGCCGACAGACTCAGGAATTGTTCCAGATATTGTGGTCGACGAAGATTTTCAGGACGAA
                                                         *

30:MITF-201       CCAAAAACGTATTACGAATTGAAGAGTCAGCCCTTGAAAAAAAACAGTAAACCTTCAGAC
28:MITF-201       CCAAAAACGTATTATGAATTGAAGAGTCAGCCCTTGAAAAAAAACAGTAATCCGTTAGAC
16:mitfa-201      ----------------------------AGCCTCTG------------------------
14:mitfa-201      TCCAAAACGTTTAACGAGTTGAAAAGCAAGCCCTTG---AAAAGCAATGATCTTCTGGAC
                                              ****  **

30:MITF-201       CAGCCATGCTC-------ATCTTCCAAGCCCCCATTGGGCTCCTCTACCACGACATCACG
28:MITF-201       CAGCAATGCTC-------ATCTTCCAAGCCCCCGTTGGGCTCTTCCGCCATGACATCACG
16:mitfa-201      -----------------------------------TGTGCCCC-CCGTGGTGACGTCACG
14:mitfa-201      CAACATGGGTTCCCCAAGCCCCCCAGAGCCCCACAGTGCTCC-CCGCAGTGACGTCACG
                                                      * ** *  *      *** *****

30:MITF-201       GATCCTGCTGCGGCAGCAGTTAATGCGAGAGCAGCTCCAGGAGCAGGAGCGGCGGGAGCA
28:MITF-201       AATCCTGCTGCGGCAGCAGTTGATGCGAGAGCAGCTCCAGGAGCAGGAGCGGCGGGAGCA
16:mitfa-201      CGTCCTTCTCTGCCAGCAGCTGATGTGGGAACAGCTCCAGGAGCAGGAGCATCGGGAACA
14:mitfa-201      TGCCCTGCTCCACCAGCAGCTGATGCAGGACCAGCTCCAGGAGCAGGACCAGCGGGAACA
                     *** **    ****** * ***   ** ***************** *  ***** **

30:MITF-201       G------CAGAGA--------CGTCAGGCCTCCTCCACCTCGCAGTACCCCCAGATCACC
28:MITF-201       G------CAGAGA--------CGTCAGGCCTCCTCCACCTCGTTGCACCACCAGACCACC
16:mitfa-201      GGGGGAACAGGGAGAGAGGAGCACCAGGCACC-----CCTCACACCACTTTCTGCCCTTC
14:mitfa-201      GAGGGAACAGAGAGAGAGAGGAGCACCAGGCACC-----CCTCACACCATCTCCTGCACCCC
                  *      *** **        *  *****  *     ****     *    * *  *  *

30:MITF-201       GCCGCCCACAATCACACGCACGGCCAGACACCGGCCATCAACGTCAGCGCTCCTCCCAGC
28:MITF-201       GACGGCCACATCCACAC------CCAGACCCCGGCCATCAACGTCAGCGCGCCCCCCAGC
16:mitfa-201      AGCACCCC---------------CCAGACCCCCACCATCAATGTCACACCGTCCCCCAAC
14:mitfa-201      AGCAGCCC---------------CCAGACCCCTCCCATCAATGTCACGCTGCCCTCCAAC
                    *  **                ****** **  ******* ****      *  *** *

30:MITF-201       CTGCCCCCCGCATCCCAGCTGCCCATGGAGGTGCTGAAGGTGCAAACTCACCTGGAGAAC
28:MITF-201       CTTCCCCCCGCATCCCAGCTGCCCATGGAGGTGCTGAAGGTGCAGACTCACCTGGAGAAC
16:mitfa-201      CTGCCCGCTGTGACCAATGTGTCCATGGAA---------GTGCAGACCCATCTGGAGAAC
14:mitfa-201      CTGCCCACTGTGACCCATGTGCCCATGGAAATCCTCAAGGTACAGACGCATCTGGAGAAC
                  ** *** * *   ** *  ** *******          ** ** ** ** *********
GAGAACCCCACCAAGTACCA
30:MITF-201       CCCACCAAGTACCACATCCAGCAGGCCCAGCAGCAGCAGGTGAAGCGCTACCT-------
28:MITF-201       CCCACCAAGTACCACATCCAGCAGGCCCAGCAGCAGCAGGTGAAGCGCTACCT-------
16:mitfa-201      CCCACCAAGTACCACATTCAGCAGGCCCAAAGACAGCAGGTGAGGCAGTACCTGTCCACC
14:mitfa-201      CCCACCAAGTATCACATCCAGCAGGCCCAGAGGAAGCAGGTGAGGCAGTACCTGTCCACA
                  *********** ***** ***********     ********* **  *****

30:MITF-201       --------GGGCAAGATTGGCTCCCTGCCC------------TGCCCTAACCAGCCCGCT
28:MITF-201       --------GGGCAAGCTTGGCTCCCTGCCC------------TGCCCCAACCAGTCCTCT
16:mitfa-201      ACCCTCGGGGGTAAGCCTGGCAGCCAGGCCAGCAGCCTGCAGTGCCACAGCCAGCCCCCG
14:mitfa-201      ACCCTTGGGGGTAAGCCTGGCAGCCAGGCCGGCAGCCTACAGTGCCCCAGCCAGCCCCCG
                          *** ***  ****  ** * **            ****  * **** ** *

30:MITF-201       GACCACGGGGGCATGCCTCCGGGGCCGGGCAACAGTGCCCCCAACAGCCCCATGGCCTTA
28:MITF-201       GACCACAGGGGCATGGCTCTGGGGCCGGGCAACAGTGCCCCCAACAGCCCTATGGCCTTA
16:mitfa-201      GAGCACGGG---ATGCCCCCCGGCCCAGGCAGCAGTGCCCCCAACAGCCCCATGGCCCTG
14:mitfa-201      GAGCACGGG---ATGCCCCCCGGCCCAGGCAGCAGTGCCCCCAACAGCCCCATGGCCCTG
                  ** *** **   *** * *  ** ** **** *****************  ****** *

30:MITF-201       CTCACCCTCAACATCAACTGCGAGAAAGAGATGGATGATGTCATTGATGACATAATTAGT
28:MITF-201       CTCACCCTCAACATCAACTGTGAGAAAGAGATGGATGATGTCATTGATGACATAATTAGT
16:mitfa-201      CTGACCCTCACCTCAAACTGCGAGAAGGAGATGGACGATGTCATTGATGACATTATTAGC
14:mitfa-201      CTGACCCTCAGCTCCAACTGCGAGAAGGAGATGGATGATGTCATTGATGACATTATTAGC
                  ** ******* *   ***** ***** ******** ***************** *****

30:MITF-201       CTGGAATCTAGTTATAACGATGATATCCTTGGATTAATGGACCCACGGCTTCAGATGGCC
28:MITF-201       CTGGAATCGAGTTATAACGACGATATCCTTGGATTAATGGACCCAGGGCTTCAGATGGCC
16:mitfa-201      TTGGAAACAAGTTATAATGATGATATTCTTGGATTTATGGACCCCGGCCTCCAGATTACA
14:mitfa-201      TTGGAAACAAGTTATAATGAGGATATTTTTGGACTTATGGACCCAGGACTCCAGGTTACT
                   ***** * ******** ** *****  ***** * ********  * ** *** *  *
```

```
30:MITF-201        AATACGATCACTGTCAACACTAACCTCTTGGAACTGTATGGTAACCAGGGCATGCCCCCT
28:MITF-201        AATACGATCGCTGTCAACACTAACCTCTTGGATATTTATGGTAACCAGGGCATGCCCCCT
16:mitfa-201       AATACACTCCCTATATCTGGTAACCTTCTGGACATGTATGGAAACCA-------------
14:mitfa-201       AATACACTCCCTGTATCTGGTAACCTTCTGGACATGTATGGAAATCAAGGGCTTCCCCCA
                   *****  ** ** *      ******  ****  * ***** ** **

30:MITF-201        CCAGGACTGGCCATC---AACTCCTGCCCTGCCAACTTGCCCAACATCAAAAGGGAATAC
28:MITF-201        CCAGGCCTGGCCATC---AACTCCTGCCCTGCCAACCTGCCCAACATCAAAAGGGAATAC
16:mitfa-201       --CGGACTTACCAGCAGTAACTCCTGCCCTGGTAGTTTATCCAACATAAAAAGGGAATTC
14:mitfa-201       AGTGGACTTGCCATCAGTAACTCCTGCCCTGGTAGCTTATCCAACATCAAAAGGGAATTC
                      ** **  *** *   *************  *   *  ******* ********** *

30:MITF-201        TCAGTTTCCCAATCTCCGGCCATTATGCACA---TGGACAAGTCAGAATCATGTGGCAAG
28:MITF-201        TCAGTTTCCCAATCTCCAGCCATCATGCATATGCTGGACAAGTCTGGATCGTGTAGCAAG
16:mitfa-201       TCAG---------CTCCTGGCATGATGAACATACTTGACAAGACTGCATCCTGTGGCCAG
14:mitfa-201       TCAG---------CTCCTGGCATGATGCACGTACTGGACAATACTGGATCCTATGGCCAG
                   ****         **** * *** *** *     * *****  * * *** * * ** **

30:MITF-201        TTTGAGAACTATCAGAGACCTGAAGGGTATCCTGTAGAAGCAGAGGTCAGGGCAATGGCA
28:MITF-201        TTTGAGAACTATCAAAGGCCTGAAGGGTTTCCTGTAGAAGCAGAGGTCAGGGCAATGGCA
16:mitfa-201       TTTGACAACTACCAAAGGCCTGAGGGCTTTCCAGTTGAAGCTGAGGTCCGAGCGATGGCC
14:mitfa-201       TTTGACAACTACCAAAGGCCCGAGGGCTTTCCAGTT---GCTGAGGTTCGAGCGATGGCC
                   ***** ***** ** ** ** ** ** * *** **    ** *****  * ** *****

30:MITF-201        AAGGAACGACAGAAGAAGGATAACCATAATTTGATTGAGAGAAGACGGAGGTTTAACATC
28:MITF-201        AAGGAACGACAGAAGAAGGATAACCATAATTTGATTGAGAGAAGACGGAGGTTTAACATC
16:mitfa-201       AAGGAGAGACAAAAAAGGACAACCACAACTTAATTGAACGAAGAAGGAGGTTCAACATC
14:mitfa-201       AAAGAGAGACAAAAAAAGGACAACCACAACTTAATTGAACGAAGGAGAAGGTTCAACATC
                   ** **  **** ** ***** ***** ** ** *****  ****  * ***** ******

30:MITF-201        AATGACCGGATCAAAGAATTGGGAACTATGATTCCAAAATCAAGTGATCCGGATATGCGT
28:MITF-201        AACGACCGGATCAAAGAATTGGGGACCATGATTCCTAAATCAAATGATCCGGATATGCGT
16:mitfa-201       AACGATCGAATCAAAGAGCTTGGAACCTTGATTCCTAAGTCAAATGATCCGGACATGCGC
14:mitfa-201       AACGATCGAATCAAAGAGCTTGGAACCCTGATTCCTAAGTCTAATGATCCGGACATGCGC
                   ** ** ** ********  * ** **  ******* ** ** * ********* *****

30:MITF-201        TGGAACAAAGGCACCATTCTCAAGGCGTCGGTGGAGTACATCAGGAAGCTGCAGCGGGAG
28:MITF-201        TGGAACAAAGGCACCATTCTCAAGGCATCAGTGGACTACATCAGGAAGCTACAGAGGGAG
16:mitfa-201       TGGAATAAGGGCACCATTCTGAAGGCCTCGGTGGACTACATCAGGAAGCTGCAGAGGGAG
14:mitfa-201       TGGAATAAGGGCACCATTCTGAAGGCCTCAGTGGACTACATCAGGAAGCTGCAGAGGGAG
                   ***** ** *********** ***** ** ***** ************** *** *****

30:MITF-201        CAACAGGGGGCCAAAGAGCTGGAGAACAGGCAGAAGAAGCTGGAGCACATCAATAGACAC
28:MITF-201        CAGCAGAGGGCCAAAGAGCTGGAGAACAGGCAGAAGAAGCTGGAGCACGCCAATAGACAC
16:mitfa-201       CAGCAGAGAGCCAAGGAGCTGGAGCTTAGACAGAGGAGGCTGGAGCATGCAAATCGGCAT
14:mitfa-201       CAGCAGAGAGCTAAGGAGGTGGAGCTTAGACAGAGAAGGCTGGAGCATGCCAACCGCCAT
                   ** *** * ** ** *** *****   ** ****  * *********    **  * **

30:MITF-201        CTGATGATGCGGATACAGGAGTTGGAGATGCAGGCTCGTGCCCATGGTCT---GACCACA
28:MITF-201        CTGATGCTGCGAATACAGGAGTTGGAGATGCAAGCTCGTGCCCATGGTCT---GACCACA
16:mitfa-201       CTGCTGCTGCGCATACAGGAGTTGGAGATGCAGGCACGGGCTCATGGTCTTGCGGTTGTG
14:mitfa-201       CTGATGCTGCGCATACAGGAGTTGGAGATGCAGGCACGGGCTCATGGTCTTGCGATTCTG
                   *** ** **** ******************** ** ** ** ********   *

30:MITF-201        GACTCGTCTGACCTCTGCTCAGCGGAGCTCTCAGCCCGAGGCATCAAGCAGGAGCCAGCC
28:MITF-201        GACACATCTGCCCTCTGCTCAGCTGAGCTCTCAGCCCGAGGCATCAAGCAGGAGCCAGCC
16:mitfa-201       CCGTCCCCTTCCCTCTGCTCCTCTGAGCTGATGGCCCGAGCCATCAAGCAGGAGCCCATC
14:mitfa-201       CCATCGTCTTCCCTCTGCTCCTCTGAGCTGATAGCCCGAGCCATCAAGCAGGAGCCCATC
                       *  **  *********  * *****    ******* ***************   *

30:MITF-201        CTGGGAGATTTCCACCAGGATCTGTACCCTGTCGACCCCCAGCACCAACACCACCCAGCC
28:MITF-201        CTGGGAGACTGCCACCAGGATCTGTACCATATCCACTCTCAGCACCAGCACCACCCAGCC
16:mitfa-201       CTAGGAGACTGTCCGTCAGACCTGTAC---------------------CAGAAGTCAGGC
14:mitfa-201       TTAGGAGACTGTCCCTCAGACCTGTAC---------------------CAGCAGCCAGGT
                    * ***** *  *     ** ******                     **  *  ***

30:MITF-201        TGCACTCCAGACCAGGTTCAGTCCACCACCTTGGAGCTCAATGATGAAACCTCTCCCTAT
28:MITF-201        TGCACTCCAGACCAGGTTCAGTACACCACCTTGGAGCTCAACGATGGAGCATCTCCCTAC
16:mitfa-201       ------CCCGACATGTCCCCTACCACCACACTAGACCTCAACAACGGCACCATCCACTTC
14:mitfa-201       ------CCCGACATGTCCCCTCCCACCACACTGGACCTCAACAACGGTACCATCCACTTT
```

```
                        ** ***  *   *    ******  * ** *****  * *   *    * **

30:MITF-201       ACCGAGGGCCACGGGGGAATCTCAGGCGAGC-----------------------------
28:MITF-201       ACCAAGGGCCACGGGGGAGTCTCAGGTGACCAGGGGCCTTACGGCGGTCATCTTAAGGGG
16:mitfa-201      AATGACAGCCCATTGGATG---CAGGGGAACCAAGGGCCTATGGC------TCCAACAAG
14:mitfa-201      AACGACAGCCCTGTGGATG---CTGGTGACCCAGGGGTATATGGC------TCCAGCAAA
                  *   *  ***    **      * ** ** *

30:MITF-201       ----------------------TCCGGATGGATGACACCTTGTCCCCGGTGGGAGGGGGA
28:MITF-201       GCCT---------TGATGGACATCCTGATGGACGACACCTTGTCCCCTGTGAGAGGGGGA
16:mitfa-201      GCCTCCACTAAACTGAAGGACAT---AATAGACAACCCCCTGTCACCCATATCA------
14:mitfa-201      GCATCCACTAAACTGAAGGACATTCTAATGGACAACACCCTGTCGCCCATATCATCCAAT
                                        *    ** **  ** ** **** **  *   *

30:MITF-201       GACCCCCTGCTCTCTTCTGTCTCGCCCGGGGCCTCTAAGGACA---GCAGCTGCTCAGGC
28:MITF-201       GACCCCCTGCTTTCCTCTGTCTCGCCCGGGGCTTCTAAGGACA---GCAGCTGCTCAGGC
16:mitfa-201      ---TCCCTTCTGTCCTCAGTGTCTCCAGACGCCTCCAACAGCAGCGGCAGCAGGCGTAGC
14:mitfa-201      GACCCCCTTCTGTCCTCAGCTTCCCCAGACACCTCCAACAGCA---GCAGCAGGCGTAGC
                      **** ** ** ** *  ** ** *   * ** **   **   ***** *     **

30:MITF-201       AGCA---TAAGCATGGAAGAGAACGACCATGGCTGTTAG
28:MITF-201       AGCA---AAAGCATGGAAGAGAACGACCAGAGCTGTTAG
16:mitfa-201      AGCAGCTCAAGCATGGAGGAGAATAATCATGGTTGTTAG
14:mitfa-201      AGCAGCTCAAGCATGGAGGAGAATGATCATGGTTGTTAG
                  ****    ********* *****  * **  * ******
```

Supplementary Figure S1: Nucleotide alignment of *mitf* paralogue coding sequences (CDS) in brown trout. Locations of paralogue specific primers are colour coded. Reverse primer used for amplification of paralogue on chromosome 30 aligns with two more paralogues; specific amplification was achieved with paralogue specific forward primer. Exon boundaries are marked with vertical lines.

```
mitfa-201        ------------------------------------------------------------ 0
14:mitfa-201     MPTDSGIVPDIVVDEDFQDESKTFNELKSKPLKSNDL--LDQHGFPKPPQSPTVLPAVTS 58
16:mitfa-201     ---------------------------------------------------WSLCVPPVVTS 11
mitfb-201        MQSESGIVPDFEVGDDFHEEPKTYYELKSQPLQ-NSNPSEQQHGSCKPPLG---SS---- 52
28:MITF-201      MQAESGIVPDFEVGEEFQEEPKTYYELKSQPLKKNSNPLDQQCSSSKPPLG---SSAMTS 57
30:MITF-201      MQAESGIVPDFEVGEEFQEEPKTYYELKSQPLKKNSKPSDQPCSSSKPPLG---SSTTTS 57

mitfa-201        ----------------------------------------------------------ML 2
14:mitfa-201     RALLHQQLMQDQLQEQDQREQREQRERSTRHP--SHHLLHPSSPQTPPINVTLPSNLPTV 116
16:mitfa-201     RVLLCQQLMWEQLQEQEHREQGEQGERSTRHP--SHHFLPFSTPQTPTINVTPSPNLPAV 69
mitfb-201        RVLLRQQLMREQLQQQERREQQKRQISI---------------THSPAINVSHPCGPPSA 97
28:MITF-201      RILLRQQLMREQLQEQERREQQRRQASSTSLHHQTT--DGHIHTQTPAINVSAPPSLPPA 115
30:MITF-201      RILLRQQLMREQLQEQERREQQRRQASSTSQYPQITAAHNHTHGQTPAINVSAPPSLPPA 117

mitfa-201        EMLEYSHYQVQTHLETPSKYHIQQSQRQQVKHYLSSALGAKLSPQASTGPGPSQPAEH-G 61
14:mitfa-201     THVPMEILKVQTHLENPTKYHIQQAQRKQVRQYLSTTLGGKPGSQAGSLQCPSQPPEH-G 175
16:mitfa-201     TNVSME---VQTHLENPTKYHIQQAQRQQVRQYLSTTLGGKPGSQASSLQCHSQPPEH-G 125
mitfb-201        AQVPMEVLKVQTHLENPTKYHIQQAQRQQVKAYLSTTLGGK---QAVSLPCPSQASDHGG 154
28:MITF-201      SQLPMEVLKVQTHLENPTKYHIQQAQQQQVKRYLGK---------LGSLPCPNQSSDHRG 166
30:MITF-201      SQLPMEVLKVQTHLENPTKYHIQQAQQQQVKRYLGK---------IGSLPCPNQPADHGG 168
                   :  .   ******.*:******:*::**: **..           :    .*  :* *

mitfa-201        MTPGPGASAPNSPMALLTL--NCEKEMDDVIEDIISLESSYSDDILGF-MDAGLQMTNTI 118
14:mitfa-201     MPPGPGSSAPNSPMALLTLSSNCEKEMDDVIDDIISLETSYNEDIFGL-MDPGLQVTNTL 234
16:mitfa-201     MPPGPGSSAPNSPMALLTLTSNCEKEMDDVIDDIISLETSYNDDILGF-MDPGLQITNTL 184
mitfb-201        MPPGPGNSAPNSPMALLTLNPNCEKEMDDVIDDIIGLESSYSDDIMGLSLDPGLQMANTI 214
28:MITF-201      MALGPGNSAPNSPMALLTLNINCEKEMDDVIDDIISLESSYNDDILGL-MDPGLQMANTI 225
30:MITF-201      MPPGPGNSAPNSPMALLTLNINCEKEMDDVIDDIISLESSYNDDILGL-MDPRLQMANTI 227
                 *  *** ************  **********:***.**:**.:**:*: :*  **::**:

mitfa-201        PVSANLLDMYSNHALPPAGVSISNSCPSSLPAVKRELSVTPSPGMMHIMDKAGPCGKFDS 178
14:mitfa-201     PVSGNLLDMYGNQGLPPSGLAISNSCPGSLSNIKREFS---APGMMHVLDNTGSYGQFDN 291
16:mitfa-201     PISGNLLDMYGNHGL-----TSSNSCPGSLSNIKREFS---APGMMNILDKTASCGQFDN 236
mitfb-201        PVSANLLDMYSNPGLPPPGISISNSCPANMTSVKREFSVTPSPALMHMMDKPSSCSKFES 274
28:MITF-201      AVNTNLLDIYGNQGMPPPGLAI-NSCPANLPNIKREYSVSQSPAIMHMLDKSGSCSKFEN 284
30:MITF-201      TVNTNLLELYGNQGMPPPGLAI-NSCPANLPNIKREYSVSQSPAIMHM-DKSESCGKFEN 285
                  :. ***::*.* .:      :  ****..:  :*** *   :*.:*:: *:     .:*:.

                                      basic                   helix        loop
mitfa-201        YQRPDGFPVEAEVRALAKERQKKDNHNLIERRRRFNINDRIKELGTLIPKSNDPDMRWNK 238
14:mitfa-201     YQRPEGFPV-AEVRAMAKERQKKDNHNLIERRRRFNINDRIKELGTLIPKSNDPDMRWNK 350
16:mitfa-201     YQRPEGFPVEAEVRAMAKERQKKDNHNLIERRRRFNINDRIKELGTLIPKSNDPDMRWNK 296
mitfb-201        YQRPEGFPVEVEVRALAKERQKKDNHNLIERRRRFNINDRIKELGTLIPKSNDPDMRWNK 334
28:MITF-201      YQRPEGFPVEAEVRAMAKERQKKDNHNLIERRRRFNINDRIKELGTMIPKSNDPDMRWNK 344
30:MITF-201      YQRPEGYPVEAEVRAMAKERQKKDNHNLIERRRRFNINDRIKELGTMIPKSSDPDMRWNK 345
                 ****:*:** .****:*******************************:****.********

                          loop             L1    L2     L3     L4
mitfa-201        GTILKASVDYIRKLQKEQQKAKELENRQKRLEHANRHLLLRIQELEMQARAHGLTVVASS 298
14:mitfa-201     GTILKASVDYIRKLQREQQRAKEVELRQRRLEHANRHLMLRIQELEMQARAHGLAILPSS 410
16:mitfa-201     GTILKASVDYIRKLQREQQRAKELELRQRRLEHANRHLLLRIQELEMQARAHGLAVVPSP 356
mitfb-201        GTILKASVDYIRKLQREQHRTKELENRQKKLEHTNRNLLLRIQELEIQARAHGLAM-ASS 393
28:MITF-201      GTILKASVDYIRKLQREQQRAKELENRQKKLEHANRHLMLRIQELEMQARAHGLTT-DTS 403
30:MITF-201      GTILKASVEYIRKLQREQQGAKELENRQKKLEHINRHLMMRIQELEMQARAHGLTT-DSS 404
                 ********:******:**:  :**:* **::*** **:*::******:*******:    :

mitfa-201        SLYSAELVARAIKQEPGMGDCTSNLYPHLP--------SPDMSRPTTLDLNNGTISYNDS 350
14:mitfa-201     SLCSSELIARAIKQEPILGDCPSDLYQ-QP--------GPDMSPPTTLDLNNGTIHFNDS 461
16:mitfa-201     SLCSSELMARAIKQEPILGDCPSDLYQ-KS--------GPDMSPTTTLDLNNGTIHFNDS 407
mitfb-201        ALCASELAARAIKQEPLLGDCSQDMYTSTH---AHPSCA-DMSRSSTLDLNNGTISFSDT 449
28:MITF-201      ALCSAELSARGIKQEPALGDCHQDLYHIHSQHQHHPACTPDQVQYTTLELNDGASPYTKG 463
30:MITF-201      DLCSAELSARGIKQEPALGDFHQDLYPVDPQHQHHPACTPDQVQSTTLELNDETSPYTEG 464
                  * ::** **.***** :**   .::*             *     :**:**: :   :..

mitfa-201        PTE-DGEPGVYDSPNKASTKLEDMLMDNTLSPVGSSDPLLSSGSPVPSNSSG-----SSS 404
14:mitfa-201     PVD-AGDPGVYGS-SKASTKLKDILMDNTLSPISSNDPLLSSASPDTSNS-SSRRSSSSS 518
16:mitfa-201     PLD-AGEPRAYGS-NKASTKLKDII-DNPLSPIS---SLLSSVSPDASNSSGSRRSSSSS 461
mitfb-201        HLS---------DT--HAAKLDDILMDETLTSGATNESLIS----AASNEST--HKDSMN 492
28:MITF-201      HGGVSGDQGPYGGH--LKGALMDILMDDTLSPVRGGDPLLSSVSPGASKDSS--CSGSKS 519
30:MITF-201      HGGISGE----------------LRMDDTLSPVGGGDPLLSSVSPGASKDSS--CSGSIS 506
                                          :  *: *:       *:*      *:.       * .

mitfa-201        MDEHDNGC     412
14:mitfa-201     MEENDHGC     526
16:mitfa-201     MEENNHGC     469
mitfb-201        MEENQHAC     500
28:MITF-201      MEENDQSC     527
30:MITF-201      MEENDHGC     514
                 *:*:::.*
```

Supplementary Figure S2: Amino acid alignment. Brown trout (mitf from Ch:14, Ch:16, Ch:28 and Ch:30) and zebrafish (*D. rerio*; mitfa and mitfb) mitf proteins derived from duplicated gene copies were included in the alignment. The main conserved mitf domains are marked.

2. Pairwise genetic distances and dN/dS

Pairwise genetic distance between brown trout paralogues, calculated in Mega X [4] applying Kimura-2-parameter model for cds and Poisson model for amino acid and partial deletion for gap treatment, are presented in Supplementary Table S1. Interestingly, nucleotide and amino acid distance do not coincide; while nucleotide distance is smaller between paralogues from Ch:14 and Ch:16 than between Ch:28 and Ch:30, amino acid distance points to highest similarity between Ch:28 and Ch:30. More nonsynonymous mutations were generated in *mitfa* gene copies (Ch:14 and 16) than in *mitfb* gene copies (Ch:28 and 30) after Ss4R duplication in brown trout (ratio of nonsynonymous versus synonymous mutations (dN/dS) is higher in *mitfa* gene copies).

Two mechanism could be considered as regulatory fate for gene duplicates in case of *mift* gene, when all four paralogues are expressed and according to protein structure retained functionality: subfunctionalization or neofunctionalization. Under neofunctionalization, one copy retains its ancestral functions, and the other acquires a novel function [5,6]. Under subfunctionalization, mutations damage different functions of each copy, such that both copies are required to preserve all ancestral gene functions. In neofunctionalization, adaptation is considered as the most distinguishing feature of this mechanism[7]; the most unambiguous evidence for adaptive evolution is an excess of nonsynonymous mutations per nonsynonymous site to synonymous mutations per synonymous site[8]. Our data do not entirely support the neofunctionalization. Excess of synonymous mutations were detected in *mitfa* and *mitfb* paralogues, but there is considerable difference in ratio of nonsynonymous versus synonymous mutations in paralogue copies of *mitf* gene in brown trout, suggesting their different evolution, selection pressure and potential neofunctionalization. Higher ratio of nonsynonymous mutations in *mitfa* paralogues, including paralogue on chromosome 14, might indicate these copies serve additional novel functions, as suggested for paralogue Ch:14 in subtype 2 erythrophores in red spots on brown trout skin.

Supplementary Table S1: Pairwise distance between brown trout *mitf* paralogues, nucleotide distance bellow and amino acid distance above diagonal.

	Ch:14	Ch:16	Ch:28	Ch:30
Ch:14		0,138	0,416	0,421
Ch:16	0,064		0,414	0,409
Ch:28	0,275	0,274		0,101
Ch:30	0,329	0,320	0,115	

3. *mitf* gene tree

Four paralogues of *mitf* are present in all salmonid species with genomes deposited in ENSEMBL (release 104, May 2021), two copies of *mitfa* and two copies of *mitfb*. Gene tree of orthologues of brown trout *mitfa* and *mifb* and alignments are presented in Supplementary Figure S3 and S4, respectively. Gene trees were drawn using ENSEMBL.

Orthologue gene comparison revealed different divergence when orthologues of four *mitf* paralogues were compared. Brown trout paralogue Ch:16 shares from 87,7 % (with *Oncorhynchus kisutch*) to 94 % (with *O. tschawytscha*) identity with Salmonidae orthologues, paralogue Ch:14 from 88,2 % (with *O. tschawytscha*) to 95 % (with *O. kisutch*), while sequence identity of orthologues gene to brown trout paralogues on Ch:30 and Ch:28 are much higher (96,6 to 99,4%). Almost the entire sequences are aligned on gene tree presenting relationship among *mitfb* orthologues, while in *mitfa* orthologues alignment much more gaps are present. Paralogues of *mitfb* are much more conserved than of *mitfa*. Duplicated gene copies apparently evolved differently, but as presented above, retained their function, supporting the hypothesis of neofunctionalization of duplicated gene copies.

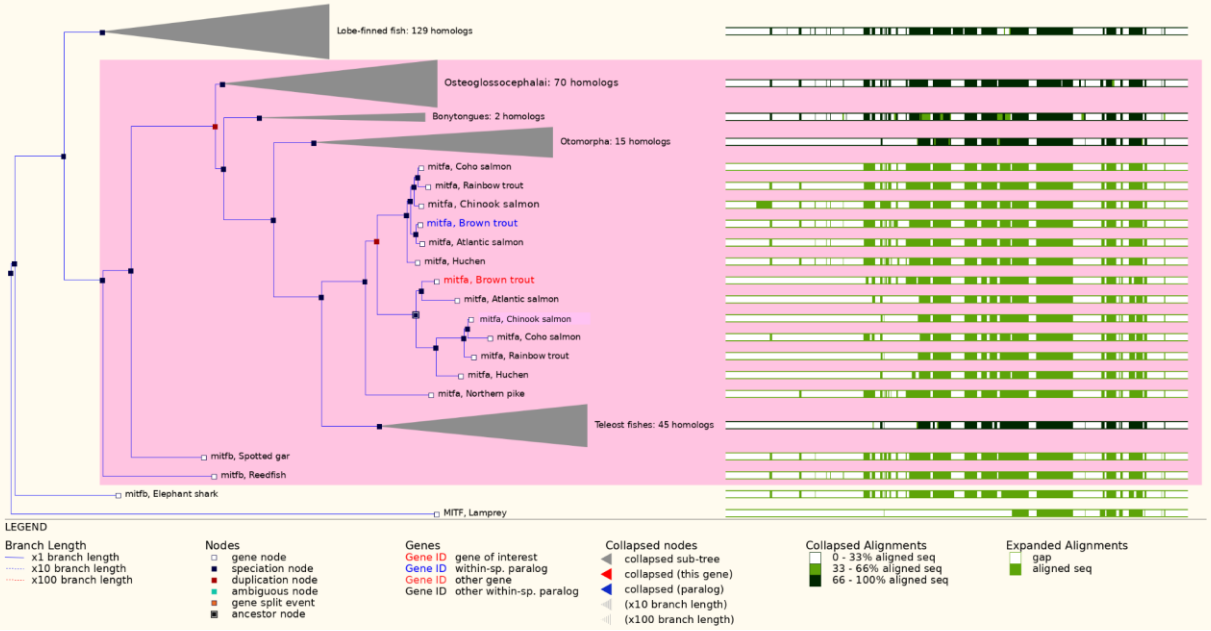

Supplementary Figure S3: gene tree of *mitfa* orthologues in fish, with expanded branches within Salmonidae, and alignments. Red coloured name denote the position of paralogue from brown trout Ch:16, blue coloured name the position of paralogue from brown trout Ch:14 on gene tree.

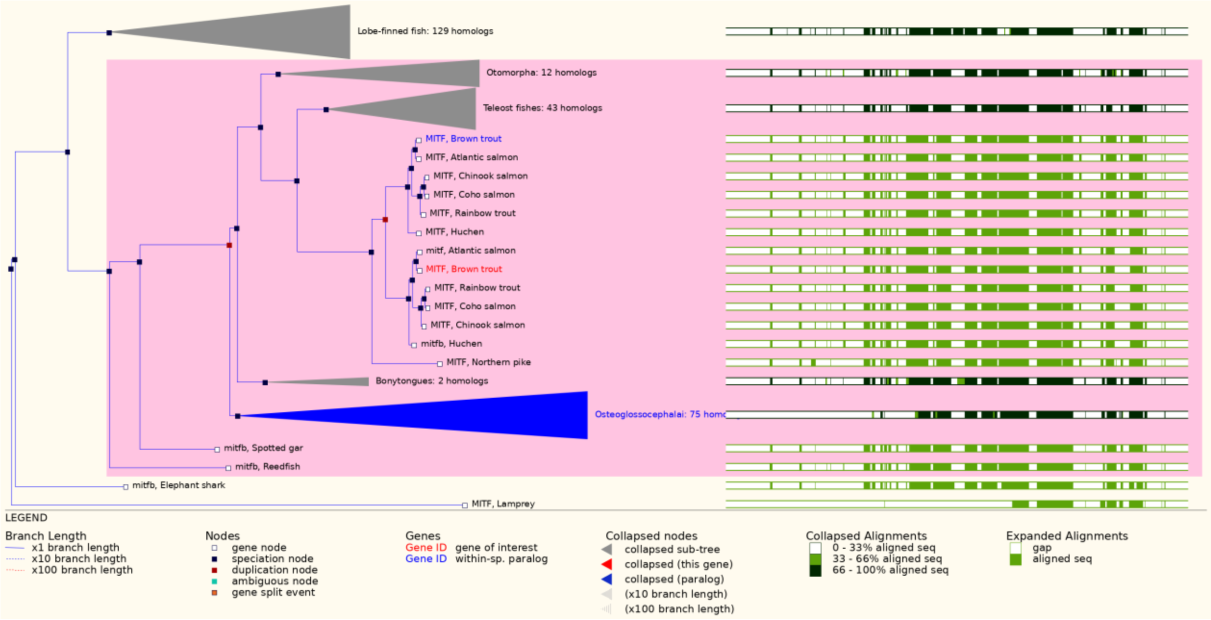

Supplementary Figure S4: gene tree of *mitfb* orthologues in fish, with expanded branches within Salmonidae, and alignments. Red coloured name denote the position of paralogue from brown trout Ch:30, blue coloured name the position of paralogue from brown trout Ch:28 on gene tree.

References

1. Djurdjevič, I., Furmanek, T., Miyazawa, S. & Sušnik Bajec, S. Comparative transcriptome analysis of trout skin pigment cells. *BMC Genomics* **20,** 1–15 (2019).

2. Madeira, F. *et al.* The EMBL-EBI search and sequence analysis tools APIs in 2019. *Nucleic Acids Research* **47,** W636–W641 (2019).

3. Hallsson, J. H., Haflidadóttir, B. S., Schepsky, A., Arnheiter, H. & Steingrímsson, E. Evolutionary sequence comparison of the Mitf gene reveals novel conserved domains. *Pigment Cell Research* **20,** 185–200 (2007).

4. Kumar, S., Stecher, G., Li, M., Knyaz, C. & Tamura, K. MEGA X: Molecular evolutionary genetics analysis across computing platforms. *Molecular Biology and Evolution* **35,** 1547–1549 (2018).

5. Ohno, S. *Evolution by gene duplication*. (Springer US, 1970).

6. Assis, R. & Bachtrog, D. Neofunctionalization of young duplicate genes in Drosophila. *Proceedings of the National Academy of Sciences of the United States of America* **110,** 17409–17414 (2013).

7. Clément, Y., Tavares, R. & Marais, G. A. B. Does lack of recombination enhance asymmetric evolution among duplicate genes? Insights from the Drosophila melanogaster genome. *Gene* **385,** 89–95 (2006).

8. Hahn, M. W. Distinguishing among evolutionary models for the maintenance of gene duplicates. *Journal of Heredity* **100,** 605–617 (2009).
